# Baicalein ameliorates cognitive decline induced by chronic cerebral hypoperfusion through the SIRT1-mediated Notch1 pathway to improve angiogenesis and suppress neuroinflammation

**DOI:** 10.3389/fnagi.2025.1521353

**Published:** 2025-04-11

**Authors:** Meixi Li, Jiaxi Song, Xiaoli Niu, Feng Mo, Xiaohua Xie, Xiuqin Li, Yu Yin, Tianjun Wang, Xiujuan Song, Jingze Liu, Peiyuan Lv

**Affiliations:** ^1^Postdoctoral Innovation Practice Base of Hebei General Hospital, Shijiazhuang, Hebei, China; ^2^Postdoctoral Research Station of Biology, Hebei Normal University, Shijiazhuang, Hebei, China; ^3^Department of Neurology, Hebei General Hospital, Shijiazhuang, Hebei, China; ^4^Hebei Key Laboratory of Cerebral Networks and Cognitive Disorders, Hebei General Hospital, Shijiazhuang, Hebei, China; ^5^Department of Rehabilitation, Hebei General Hospital, Shijiazhuang, Hebei, China; ^6^Department of Neurosurgery, Hebei General Hospital, Shijiazhuang, Hebei, China; ^7^Department of Geriatrics, Hebei General Hospital, Shijiazhuang, Hebei, China; ^8^Department of Neurology, The Second Hospital of Hebei Medical University, Shijiazhuang, Hebei, China; ^9^Hebei Key Laboratory of Animal Physiology, Biochemistry and Molecular Biology, College of Life Sciences, Hebei Normal University, Shijiazhuang, Hebei, China

**Keywords:** vascular dementia (VD), baicalein, angiogenesis, neuroinflammation, SIRT1-mediated Notch1 pathway

## Abstract

**Introduction:**

The potential for therapeutic strategies that promote angiogenesis and suppress neuroinflammation to ameliorate cognitive decline induced by chronic cerebral hypoperfusion (CCH) has led to their recognition as promising therapeutic targets for vascular dementia (VD). The SIRT1-mediated Notch1 signaling pathway is important in regulating angiogenesis and neuroinflammation. Previous studies have demonstrated that baicalein alleviates cognitive decline in rats with CCH. Nevertheless, it remains unclear whether baicalein can stimulate angiogenesis in the context of VD and whether this cognitive protective effect is achieved by regulating the SIRT1-mediated Notch1 pathway. The aim of this study was to investigate the impact and the underlying mechanism of baicalein on angiogenesis and neuroinflammation in rats with CCH.

**Methods:**

Adult Sprague-Dawley (SD) rats were administered baicalein or a SIRT1 inhibitor. Cognitive function was assessed by the Morris water maze (MWM) test, and angiogenesis was assessed by immunohistochemical analysis of microvascular density (MVD) and the number of CD31+/5-bromo-2’-deoxyuridine (BrdU)+ cells. Neuroinflammation and apoptosis were assessed by immunohistochemistry for GFAP, Iba-1, NEUN/cleaved caspase-3, and ELISA analysis for TNF-α and IL-1β. Additionally, Western blotting was employed to evaluate the expression of the SIRT1-mediated Notch1 pathway.

**Results:**

The results demonstrated that baicalein ameliorated memory and learning deficits in rats following CCH by promoting angiogenesis and suppressing neuroinflammation. However, this protective effect could be reversed by inhibiting SIRT1. Baicalein was observed to up-regulate the expression of SIRT1 and down-regulate the Notch1-related molecules.

**Discussion:**

The SIRT1-related pathway plays a crucial role in regulating angiogenesis and neuroinflammation. Moreover, baicalein exerts a neuroprotective effect against cognitive decline through the SIRT1-mediated Notch1 pathway, which in turn improves angiogenesis and suppresses neuroinflammation.

## 1 Introduction

The prevalence of dementia is expected to triple by 2050 (Iadecola, 2013). Vascular dementia (VD) is currently the second most common form of dementia surpassed only by Alzheimer’s disease. Previous studies have indicated that chronic cerebral hypoperfusion (CCH) leads to capillary loss (Marien et al., 2016), autophagy dysfunction and neuronal loss in the hippocampus (Li X. et al., 2017; Xu et al., 2017), inflammation (Li et al., 2020; Meng et al., 2020), and endoplasmic reticulum stress (Niu et al., 2019), all of which contribute to the development of vascular cognitive impairment. Angiogenesis, which primarily denotes the phenomenon of new capillary growth through sprouting from existing blood vessels, represents a compensatory response to hemodynamic changes and may serve as a potential therapeutic target for VD due to its capacity to facilitate the recovery of cognitive deficits (Ren et al., 2018; Wang and Hu, 2018). Nevertheless, the precise molecular mechanism underlying angiogenesis in CCH remains to be elucidated.

Silent mating type information regulation 2 homolog 1 (SIRT1), which is known as an NAD (+)-dependent deacetylase, has been demonstrated to be involved in a number of pathological processes including those related to metabolism, inflammation, and angiogenesis (Rogina and Tissenbaum, 2024). A previous study demonstrated that silencing SIRT1 abolished the endothelial cell proliferation, angiogenic and migratory ability (Zhao et al., 2024). Conversely, pharmacological activation of SIRT1 has been demonstrated to increase the vascular endothelial growth factor-a (VEGF-a) in protein level following focal cerebral ischemic injury (Hermann et al., 2015). The pro-angiogenic effects of SIRT1-mediated VEGF have been demonstrated in certain ischemic models (Hu et al., 2016; Zheng et al., 2018). Nevertheless, the function of the SIRT1 pathway in angiogenesis in VD remains unclear.

Recent studies have demonstrated that the delta-like ligand (DLL)-4, which is recognized as the classical ligand for Notch1, leads to a reduction in the expression of VEGF receptor (VEGFR)2 once bound to Notch1 in adjacent stalk cells. This, in turn, lowers the sensitivity of vascular endothelial cells (VECs) to VEGF. This ultimately leads to the limitation of tip cells and the prevention of excessive sprouting (Bashir et al., 2024). Inhibition of DLL4-Notch signaling has been demonstrated to up-regulate VEGFR2 and VEGFR3, thereby promoting the response to VEGF and contributing to enhanced angiogenic function (Wang et al., 2014). Additionally, the up-regulation of Notch signaling plays a significant role in the activation of microglia and the subsequent inflammatory response following an ischemic stroke (Qin et al., 2019). Moreover, the inhibition of the Notch pathway has been demonstrated to reduce NF-kB/p65 expression and translocation (Yao et al., 2013a). These results offer a potential explanation for the relationship between Notch and angiogenesis and neuroinflammation. Moreover, it has been demonstrated that SIRT1 deacetylates the intracellular domain of Notch (NICD), thereby inhibiting NICD translocation into the nucleus and subsequent gene expression (Wang et al., 2014). Accordingly, the present study was designed to assess the involvement of the SIRT1-mediated Notch1 pathway in regulating angiogenesis and neuroinflammation in rats after CCH.

Baicalein (5,6,7-trihydroxy-2-phenyl-4H-1-benzopyran-4-one) is a major flavonoid extracted from the *Scutellaria baicalensis Georgi*. It has been demonstrated to possess a range of biological functions, including anti-apoptotic properties and anti-inflammatory effects. However, its role in angiogenesis remains a topic of contention. Previous studies have demonstrated that baicalein accelerates dental pulp repair and regeneration by promoting angiogenesis (Lee et al., 2016), strengthens angiogenesis and increases blood flow to ischemic limbs in diabetic mice (Liu et al., 2023), but inhibits angiogenesis and synovial proliferation to alleviate osteoarthritis (Li et al., 2021). The findings of our research group indicate that baicalein has the capacity to mitigate the CCH-induced cognitive dysfunction by promoting remyelination (Xiao et al., 2023), suppressing neuroinflammation and regulating the composition of the intestinal microbiota (Song et al., 2024). Nevertheless, it remains unclear whether baicalein can promote angiogenesis to alleviate vascular cognitive impairment.

In light of the aforementioned research background, this study employed bilateral common carotid artery occlusion (BCCAO) to simulate the CCH-induced VD, and baicalein and a SIRT1 inhibitor were administered to investigate whether the therapeutic efficacy of baicalein was associated with the promotion of angiogenesis and the inhibition of neuroinflammation, and whether the SIRT1-mediated Notch1 pathway plays a role in promoting angiogenesis and suppressing inflammation in rats with CCH, and whether the cognitive protective property of baicalein is related to the regulation of the SIRT1-mediated Notch1 pathway.

## 2 Materials and methods

### 2.1 Animals

**FIGURE 1:**
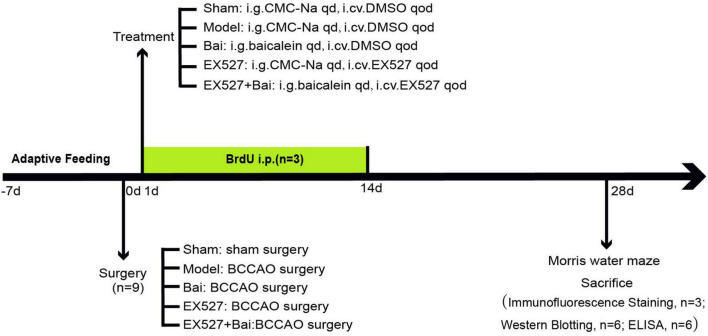
The complete flow chart of this study.

Adult male Sprague- Dawley (SD) rats (300–320 g, aged 8 weeks) were procured from Hebei Medical University (license No. SCXK(Ji) 2018-004) and maintained at a room temperature of 24 ± 2°C and a humidity of 60-70% on a 12:12-h light/dark cycle in the laboratory animal center of Hebei General Hospital. The animals had free access to food and water throughout the whole course of the study. All procedures were approved by the Ethics Committee of Hebei General Hospital (No. 202333) and conducted in accordance with the Guide for the Care and Use of Laboratory Animals of the National Institutes of Health (Bethesda, MD, United States). The experimental flowchart is displayed in Figure 1.

### 2.2 Surgical procedure

BCCAO represents a well-established methodology for the induction of CCH in rodents, as a means of modeling VD (Ren et al., 2018). Briefly, the animals were anesthetized via 3% pentobarbital sodium (i.p., 0.2 ml/kg), after which the bilateral common carotid arteries were exposed and separated gently from the surrounding tissues in a manner that avoided contact with the vagus nerve. Subsequently, the common carotid arteries were ligated on two occasions with 4- 0 silk sutures and then severed between the ligatures. Finally, the wound was meticulously sutured and disinfected. All procedures were conducted in a heated environment to maintain the animals’ body temperature at 37 ± 0.5°C. Subcutaneous injections of ketoprofen (10 mg/kg) were administered to all animals 2 h post-surgery and twice a day for 2 consecutive days to provide analgesia. The procedure was identical for sham-operated animals, with the exception of the ligation and dissociation of the bilateral common carotid arteries. Forty-five rats underwent the surgical procedure and were successfully discharged from the study. Two rats died following the surgical procedure.

### 2.3 Groups and drug administration

The sham- operated rats were assigned to the sham group, which received vehicle treatment (i.g., 0.5% sodium carboxymethylcellulose, CMC-Na, i.cv., dimethyl sulfoxide, DMSO). The remaining 36 rats were randomly assigned to four groups (*n* = 9/group) following the BCCAO surgery. The groups were as follows: (1) the model group, which underwent the BCCAO surgery and received vehicle treatment (i.g., CMC-Na, i.cv., DMSO); (2) the Bai group, which underwent the BCCAO surgery and received baicalein 100 mg/kg/d in diluent (i.g., CMC-Na) and vehicle treatment (i.cv., DMSO); (3) the EX527 group, which underwent the BCCAO surgery and received EX527 (SIRT1 inhibitor) 10 μg every 2 days (i.cv., dissolved in DMSO) and vehicle treatment (i.g., CMC-Na); and (4) the EX527+Bai group, which underwent the BCCAO surgery and received baicalein and EX527. EX527/ baicalein was administered at 2-day intervals/once a day (between 10:00 and 12:00 a.m.) for a period of 4 weeks, commencing on the day following the establishment of the model. The dose of baicalein was chosen based on previous studies of our group in which baicalein significantly improved cognitive deficits and showed neuroprotective effects (Song et al., 2024; Xiao et al., 2023).

### 2.4 BrdU injection

To evaluate neogenesis of VECs, three rats from each group were administered 5-bromo-2′-deoxyuridine (BrdU) (i.p., 50 mg/kg) for 14 days starting 24 h after BCCAO.

### 2.5 Morris water maze test

Four weeks following the surgical procedure, the spatial learning and memory capabilities of the rats were assessed using the Morris water maze (MWM). The MWM was a black and circular pool with a diameter of 160 cm and a depth of 45 cm. The pool was filled with water, and a platform was placed 1-2 cm below the surface of the water. The MWM test comprised 2 phases as previously described (Li et al., 2020): the place navigation phase, which was conducted over 5 consecutive days, and the spatial probe phase, which was conducted on the 6th day. During the place navigation phase, each rat was placed in the pool four times from different quadrants and permitted to search for the platform. Concurrently, the escape latency was documented with the aid of a built-in camera and accompanying software. In the event that a rat was unable to locate the platform within the allotted 120 s, it was guided to the platform to learn to remember the location for 20 s, and the escape latency was recorded as 120 s. On the day of the spatial probe test, the platform was removed. The rats were placed in the pool in the quadrant diagonal to the quadrant where the platform was originally placed and allowed to search for the platform for 120 s. The frequency of crossing the platform was recorded.

### 2.6 Immunofluorescence staining

Following the MWM test, the three rats that have previously been administered BrdU were sacrificed for immunofluorescence staining. As previously described (Li et al., 2020), following anesthesia, the rats were perfused transcardially with cold 0.9% saline and 4% paraformaldehyde. After decollation, the brain was carefully removed and immersed in 4% paraformaldehyde for a period of 24 h. Tissues containing the hippocampus were embedded in paraffin and subsequently sectioned along the coronal plane (a thickness of 5 μm). After deparaffinization and subsequent antigen retrieval with EDTA (pH 8.0) in a microwave, the sections were blocked by bovine serum albumin for 30 min. The sections were then incubated at 4°C overnight with rabbit primary antibodies against CD31 (1:500, ab182981, Abcam, Cambridge, United Kingdom), GFAP (1:400, 16825-1-AP, Proteintech Biotechnology, Wuhan, China), Iba-1 (1:500, GB115173, Servicebio, Wuhan, China), NEUN (1:500, GB11138, Servicebio, Wuhan, China) and a mouse primary antibody against cleaved caspase-3 (1:300, 66470-2-Ig, Proteintech Biotechnology, Wuhan, China). For BrdU staining, DNA denaturation (1 mM HCl at 37°C) for 30 min and HCl neutralization (50 mM sodium borate buffer, pH 8.5) for 20 min were performed, followed by overnight incubation with a mouse primary antibody against BrdU (1:100, ab8152, Abcam, Cambridge, United Kingdom). On the next day, the sections were washed three times with PBS and then incubated with fluorescent-conjugated anti-rabbit (CY3-, 1:300, GB21303, Servicebio, Wuhan, China) and anti-mouse (488-, 1:400, GB21301, Servicebio, Wuhan, China) secondary antibodies for 50 min in the dark. Finally, the sections were incubated with DAPI for 10 min and washed three times again before being sealed with fluorescent mounting medium. A fluorescence microscope (Eclipse C1, Nikon, Tokyo, Japan) was used to detect fluorescent signals at a consistent exposure time. Digital images of four areas of the bilateral hippocampal CA1 region from each section were captured through a 40 × objective with an imaging system (DS-U3, NIKON, Tokyo, Japan). The microvascular density (MVD) was determined by immunofluorescence staining of CD31 and analyzed via Image-Pro Plus v6.0 software (Li Y. et al., 2017). The number of CD31^+^/ BrdU ^+^ cells, GFAP^+^ cells and Iba-1^+^ cells were counted manually by two investigators who were blinded to sample identity in order to eliminate any potential bias. The counting rules of CD31^+^/ BrdU ^+^ cells were as follows: every CD31/BrdU double-positive object, regardless of size, was counted, except for those suspected to be CD31-positive inflammatory cells (Marien et al., 2016).

### 2.7 Western blotting

Six rats were chosen randomly for western blot analysis 4 weeks after BCCAO. These rats were decapitated under anesthesia. Then, the hippocampus was dissected meticulously on an ice-cold plate and homogenized in RIPA buffer (0 °C, R0010, Solarbio, Beijing, China) in accordance with the manufacturer’s instructions. Subsequently, the tissue homogenates were centrifuged (4°C, 12,000 × g, 10 min) to obtain the supernatant, which is the total protein. For the purpose of quantifying NICD, nucleoproteins were extracted with the Nuclear Protein Extraction Kit (R0050, Solarbio, Beijing, China), in accordance with the manufacturer’s instructions. The concentrations of the protein samples were determined with the BCA Protein Assay kit (PC0020, Solarbio, Beijing, China). The steps of western blotting are as described previously (Li et al., 2020). An equal quantity of protein (40 μg) from each sample was separated on 7.5 or 10% sodium dodecyl sulfate-polyacrylamide electrophoresis (SDS-PAGE) gels and electrotransferred to polyvinylidene fluoride (PVDF) membranes. These membranes were blocked in 5% fat-free milk for 1 h and then incubated overnight at 4°C with the following primary antibodies: anti-angiopoietin-1 (Ang-1, 1:400, 23302-1-AP, Proteintech, Wuhan, China), anti-CD31 (1:500, YT0751, ImmunoWay, Plano, TX, United States), anti-VEGF-a (1:800, ab46154, Abcam, Cambridge, United Kingdom), anti-SIRT1 (1:400, ab189494, Abcam, Cambridge, United Kingdom), anti-VEGFR2 (1:5,000, 26415-1-AP, Proteintech, Wuhan, China), anti-DLL4 (1:400, GB115572, Servicebio, Wuhan, China), anti-NICD (1:500, GB111690, Servicebio, Wuhan, China), anti-Histone H3 (1:1,000, GB11102, Servicebio, Wuhan, China) and anti-β-actin (1:4,000, 20536-1-AP, Proteintech, Wuhan, China). After washing three times with TBST, the membranes were incubated for 1 h with secondary antibody (HRP-conjugated goat anti- rabbit IgG H&L, 1:5,000, ab6721, Abcam, Cambridge, United Kingdom). Afterwards, the protein bands on the membranes were visualized using an enhanced chemiluminescence (ECL) kit (WLA003, Wanleibio, Shenyang, China) and quantified using Image J analysis software. The relative intensity of each band was normalized to that of the β-actin or Histone H3 band.

### 2.8 Enzyme-linked immunosorbent assay

Six rats were chosen randomly for the purpose of determining the levels of TNF-α and IL-1β by means of enzyme-linked immunosorbent assay (ELISA). The hippocampus tissue was homogenized in PBS (0°C) and centrifuged (4°C, 3,000 × g, 20 min). The resulting supernatant was then collected to assessed the levels of TNF-α and IL-1β in accordance with the instructions provided with the ELISA kit (Servicebio, Wuhan, China).

### 2.9 Statistical analysis

All data are presented as the mean ± standard deviation and were analyzed using the IBM SPSS Statistics 20.0 software package (IBM, Armonk, New York, United States). One-way analysis of variance (ANOVA) was employed for intergroup comparisons. Once the assumption of homogeneity of variance had been verified, the Tukey Honest Significant Difference (Tukey HSD) test was employed. In instances where homogeneity of variance was not confirmed, the Tamhane’s T2 test was employed. Intragroup comparisons of escape latency were conducted using a repeated-measures ANOVA. *p* < 0.05 was considered statistically significant.

## 3 Results

### 3.1 Baicalein alleviates learning and memory deficits in rats with CCH

**FIGURE 2:**
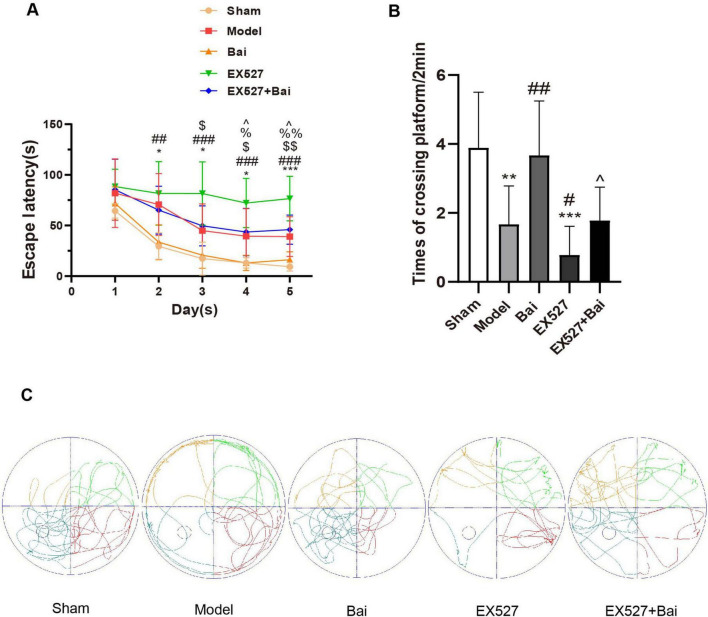
Baicalein alleviates learning and memory deficits in rats with CCH (MWM test, *n* = 9 in each group). **(A)** Variations in escape latency from day 1 to 5 in the different groups. *p < 0.05, ***p < 0.001: the model group vs. the sham group; ^##^*p* < 0.01, and ^###^*p* < 0.001: the EX527 group vs. the sham group, ^$^*p* < 0.05, and ^$$^*p* < 0.01: the EX527 group vs. the model group, ^%^*p* < 0.05, ^%%^*p* < 0.01: the Bai group vs. the model group, ^∧^*p* < 0.05: the Bai group vs. the EX527+Bai group. **(B)** Differences in times of crossing platform on day 6. **p < 0.01, ***p < 0.001: vs. the sham group; ^#^*p* < 0.05, ^##^*p* < 0.01: vs. the model group, ^∧^*p* < 0.05: vs. the Bai group. **(C)** Swimming paths of rats on day 6. The values are expressed as the mean ± SD.

The degree of cognitive deficits induced by CCH and the protective effect of baicalein and SIRT1 were assessed using the MWM test. At 4 weeks after BCCAO, the rats with CCH exhibited a pronounced deterioration in spatial learning and memory, which was mitigated by baicalein treatment but exacerbated by the SIRT1 inhibitor EX527. Moreover, the cognitive protective effect of baicalein was partially reversed by EX527. The spatial learning ability was reflected by the escape latency, which exhibited a gradual reduction in each group on the final days of training (*P* < 0.01) (Figure 2A). The model group exhibited prolonged escape latencies on days 2-5 (day 2: *P* < 0.05; day 3: *P* < 0.05; day 4: *P* < 0.05; day 5: *P* < 0.001) (Figure 2A) and a reduced frequency of platform crossings in comparison to the sham rats (*P* < 0.01) (Figures 2B,C). However, the escape latencies exhibited a reduction following the administration of baicalein (day 4: *P* < 0.05; day 5: *P* < 0.01, Bai group vs. model group) (Figure 2A), whereas their prolongation was observed upon the inhibition of SIRT1 (day 3: *P* < 0.05; day 4: *P* < 0.05; day 5: *P* < 0.05, EX527 group vs. model group) (Figure 2A). Similarly, the Bai group demonstrated a greater number of platform crossings on the 6th day than the model group (*P* < 0.01) (Figures 2B,C), whereas the EX527 group exhibited a reduced number of platform crossings compared to the model group (*P* < 0.05) (Figures 2B,C). Moreover, rats in the EX527+Bai group exhibited prolonged escape latencies (day 4: *P* < 0.05; day 5: *P* < 0.05) (Figure 2A) and a reduced frequency of platform crossings (*P* < 0.05) (Figures 2B,C) compared to rats in the Bai group. This suggests that the cognitive protective effect of baicalein was partially reversed by EX527. These findings confirm that baicalein mitigates cognitive dysfunction caused by CCH to some extent. However, this mitigation may be partially reversed by inhibiting SIRT1.

### 3.2 Baicalein promotes angiogenesis in the hippocampus CA1 region following CCH by regulating the SIRT1 signaling pathway

**FIGURE 3:**
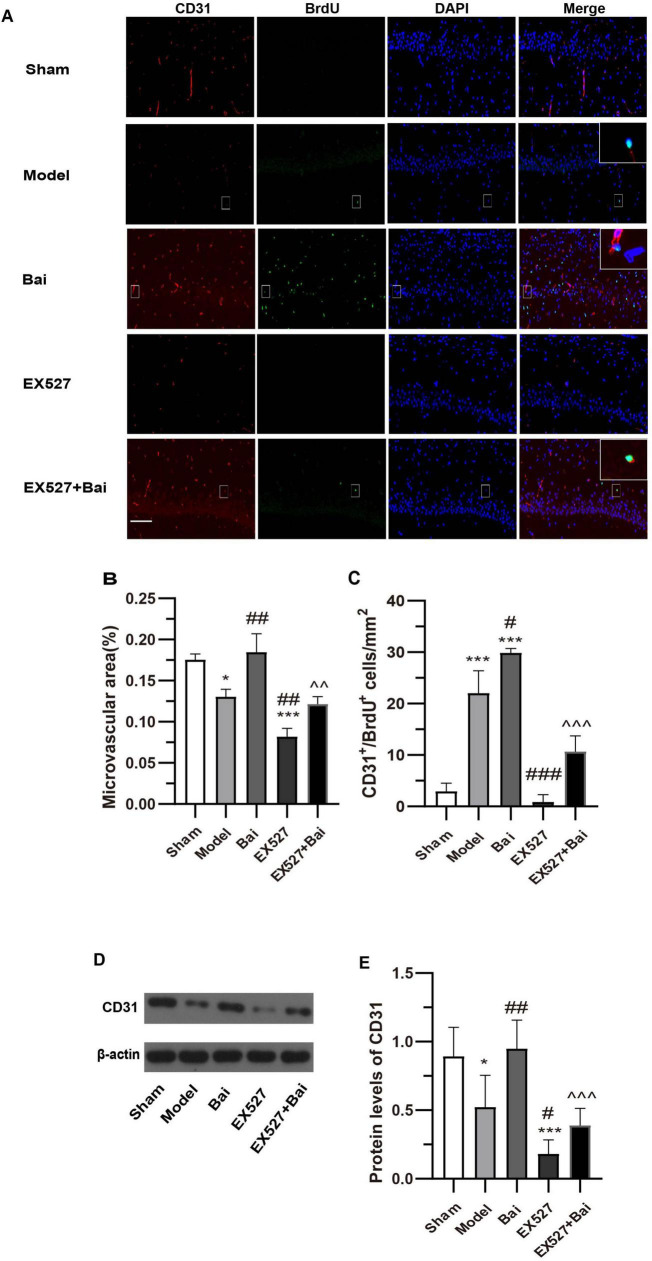
Baicalein promotes angiogenesis in the hippocampus of rats with CCH. **(A)** Representative images of CD31 (red) and BrdU (green) double immunofluorescence in the hippocampus of rats at 4 weeks post-surgery. Bar = 50 pm. Representative double-labeled areas are presented at higher magnification (*n* = 3 in each group). **(B)** The microvascular density (MVD) in the hippocampus. **(C)** The number of CD31+/BrdU+ cells in the hippocampus. **(D)** Western blot analysis of the expression of CD31 in the hippocampus (*n* = 6 in each group). **(E)** Quantitative analysis of the protein levels of CD31. β-actin was used as an internal control. *p < 0.05, ***p < 0.001: vs. the sham group; ^#^*p* < 0.05, ^##^*p* < 0.01, and ^###^*p* < 0.001: vs. the model group; ^∧∧^*p* < 0.01, ^∧∧∧^*p* < 0.001: vs. the Bai group. The values are expressed as the mean ± SD.

We employed immunofluorescence staining of CD31 to quantify MVD, which is regarded as a valuable parameter for the quantitative assessment of angiogenesis (Chen et al., 2018; Zhang et al., 2018). In comparison to the sham group, the MVD in the hippocampus CA1 region and the CD31 protein level in the model group exhibited a decrease, which can be considered a compensatory and pathological response (both *P* < 0.05) (Figures 3A,B,D,E). This finding is in accordance with those of previous studies (Marien et al., 2016). However, the MVD and CD31 protein level in the EX527 group exhibited a more pronounced decline (both *P* < 0.001, vs. sham group. MVD, *P* < 0.01; CD31 protein level, *P* < 0.01, vs. model group) (Figures 3A,B,D,E). The administration of baicalein resulted in an increase in MVD and a promotion of the protein level of CD31 (both *P* < 0.01, vs. model group), which was prevented by EX527 (MVD, *P* < 0.01; CD31 protein level, *P* < 0.001, vs. EX527+Bai group). To evaluate endothelial cell proliferation, the number of CD31+/BrdU+ endothelial cells in hippocampus CA1 region was quantified. CCH has been demonstrated to induce endothelial cell proliferation (*P* < 0.001, model group vs. sham group) (Figures 3A,C), a process that is inhibited by SIRT1 inhibition (*P* < 0.001, EX527 group vs. model group) (Figures 3A,C). In comparison to the rats in the model group, the rats treated with baicalein exhibited a greater number of CD31+/BrdU+ cells (*P* < 0.05) (Figures 3A,C). This effect was also blocked by SIRT1 inhibition (*P* < 0.001, Bai group vs. EX527+Bai group) (Figures 3A,C). These findings indicate that baicalein facilitates angiogenesis in the hippocampus following CCH, with involvement of the SIRT1 signaling pathway.

### 3.3 Baicalein suppresses neuroinflammation in the hippocampus CA1 region following CCH by regulating the SIRT1 signaling pathway

**FIGURE 4:**
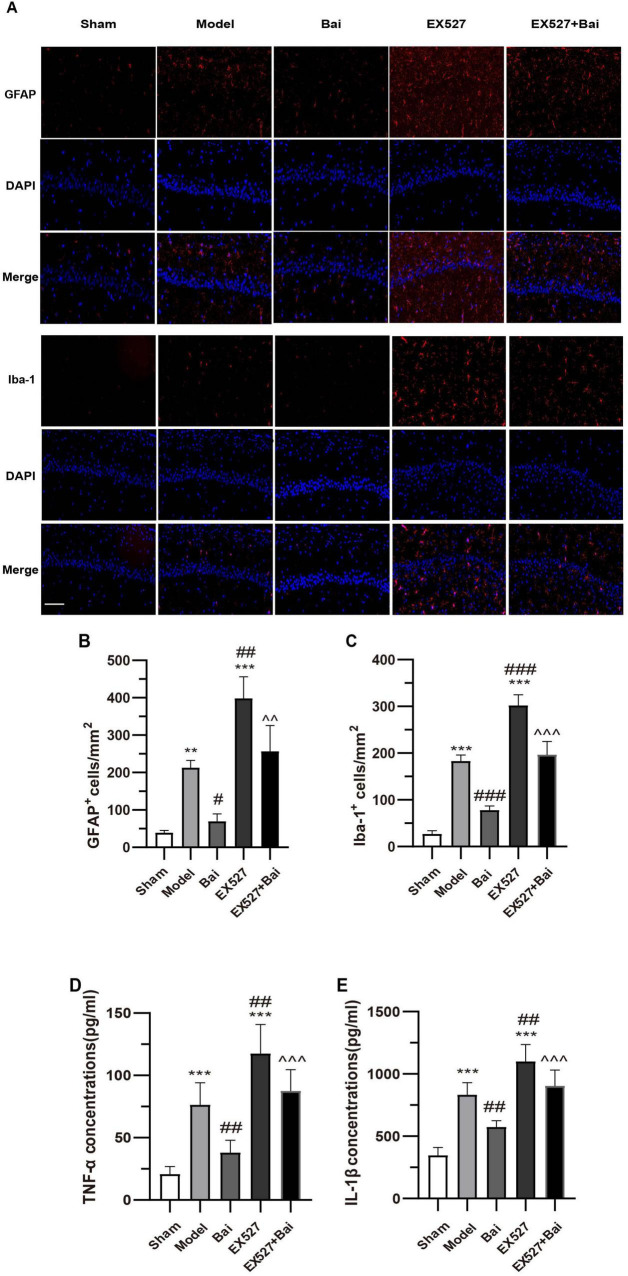
Baicalein attenuates inflammatory response in the hippocampus of rats with CCH. **(A)** Representative images of GFAP (red) and Iba-1 (red) immunofluorescence in the hippocampus (*n* = 3 in each group). Bar = 50 pm. **(B)** The number of GFAP+ cells in the hippocampus. **(C)** The number of Iba-1 + cells in the hippocampus. **(D)** Quantitative analysis of TNF-a by ELISA (*n* = 6 in each group). **(E)** Quantitative analysis of IL-1β by ELISA (*n* = 6 in each group). **p < 0.01, ***p < 0.001: vs. the sham group; ^#^*p* < 0.05, ^##^*p* < 0.01, and ^###^*p* < 0.001: vs. the model group; ^∧∧^*p* < 0.01, ^∧∧∧^*p* < 0.001: vs. the Bai group. The values are expressed as the mean ± SD.

Given the pivotal role of glial cell activation-mediated neuroinflammation in CCH-induced brain injury, the present study employed immunofluorescence staining of GFAP and Iba-1 to enumerate astrocytes and microglia, respectively, and ELISA to quantify the levels of proinflammatory cytokines (TNF-α and IL-1β). Consistent with previous research (Song et al., 2024), the present results demonstrated that CCH induces an increase in the number of GFAP^+^ and Iba1^+^ cells (GFAP^+^ cells, *P* < 0.01; Iba-1^+^ cells, *P* < 0.001, model group vs. sham group) (Figures 4A-C). It is noteworthy that the hyperproliferation of astrocytes and microglia observed in rats with CCH was reversed by baicalein treatment. This was evidenced by a significant reduction in the number of GFAP^+^ and Iba-1^+^ cells in the baicalein-treated group compared to the model group (GFAP^+^ cells, *P* < 0.05; Iba-1^+^ cells, *P* < 0.001, Bai group vs. model group) (Figures 4A-C). However, SIRT1 inhibition resulted in a further increase in the number of GFAP^+^ and Iba1^+^ cells (GFAP^+^ cells, *P* < 0.01; Iba-1^+^ cells, *P* < 0.001, model group vs. EX527 group) (Figures 4A-C). Moreover, the inhibitory effect of baicalein on the astrocyte and microglia proliferation was reversed by EX527 (GFAP^+^ cells, *P* < 0.01; Iba-1^+^ cells, *P* < 0.001, Bai group vs. EX527+Bai group) (Figures 4A-C).

As proinflammatory factors related to glial cells, TNF-α and IL-1β were elevated in the hippocampus of the model rats (both *P* < 0.001, model group vs. sham group) (Figures 4D,E). Following 4 weeks of baicalein treatment, there was a notable decrease in TNF-α and IL-1β levels (both *P* < 0.01, model group vs. Bai group) (Figures 4D,E). However, this effect could be reversed by blocking SIRT1 (both *P* < 0.01, EX527+Bai group vs. Bai group) (Figures 4D,E). These findings suggest that baicalein may exert a neuroprotective effect by reducing neuroinflammatory responses in the hippocampus of rats following CCH by regulating the SIRT1 signaling pathway.

### 3.4 Baicalein reduces apoptosis in the hippocampus CA1 region following CCH by regulating the SIRT1 signaling pathway

**FIGURE 5:**
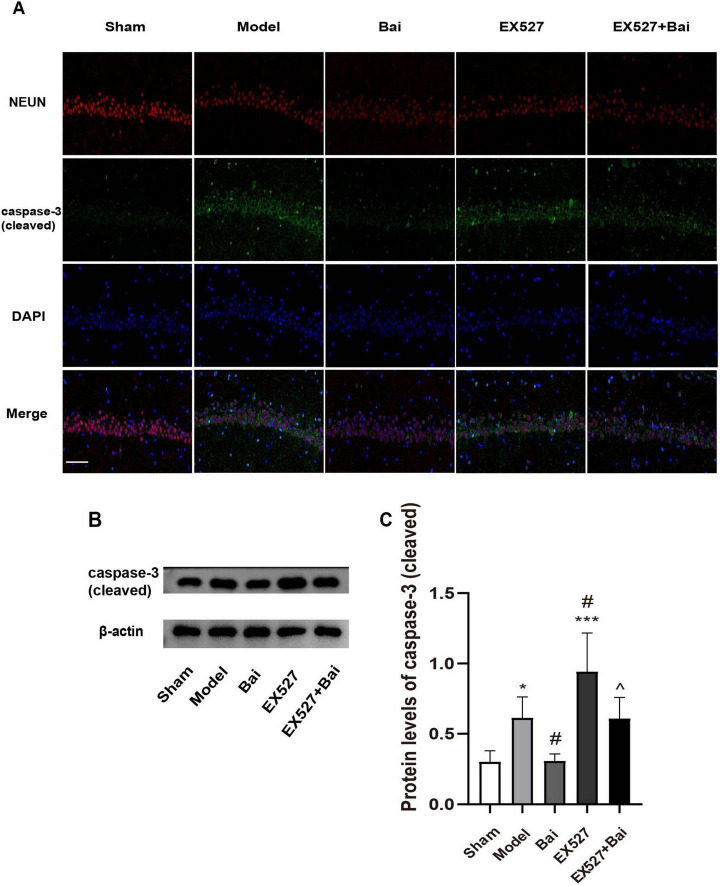
Baicalein reduces apoptosis in the hippocampus of rats with CCH. **(A)** Representative images of NEUN (red) and cleaved caspase-3 (green) double immunofluorescence in the hippocampus (*n* = 3 in each group). Bar = 50 urn. **(B)** Western blot analysis of the expression of cleaved caspase-3 (*n* = 6 in each group). **(C)** Quantitative analysis of the protein levels of cleaved caspase-3. β-actin was used as an internal control. *p < 0.05, ***p < 0.001: vs. the sham group; ^#^*p* < 0.05: vs. the model group; ^∧^*p* < 0.05: vs. the Bai group. The values are expressed as the mean±SD.

We applied immunofluorescence staining of NEUN and cleaved caspase-3, and western blot analysis of cleaved caspase-3 to reflect apoptosis in the hippocampus of rats. As illustrated in Figures 5A-C, the rats with CCH exhibited a greater incidence of hippocampal neuron apoptosis (*P* < 0.05, model group vs. sham group), which was mitigated by baicalein treatment (*P* < 0.05, model group vs. Bai group), but exacerbated by EX527 intervention (*P* < 0.001, sham group vs. EX527 group; *P* < 0.05, model group vs. EX527 group). However, the inhibitory effect of baicalein on apoptosis was reversed by EX527 (*P* < 0.05, Bai group vs. EX527+Bai group). These results suggest that baicalein reduces apoptosis in the hippocampus in rats with CCH by regulating the SIRT1 signaling pathway.

### 3.5 Baicalein regulates the SIRT1-mediated Notch1 signaling

**FIGURE 6:**
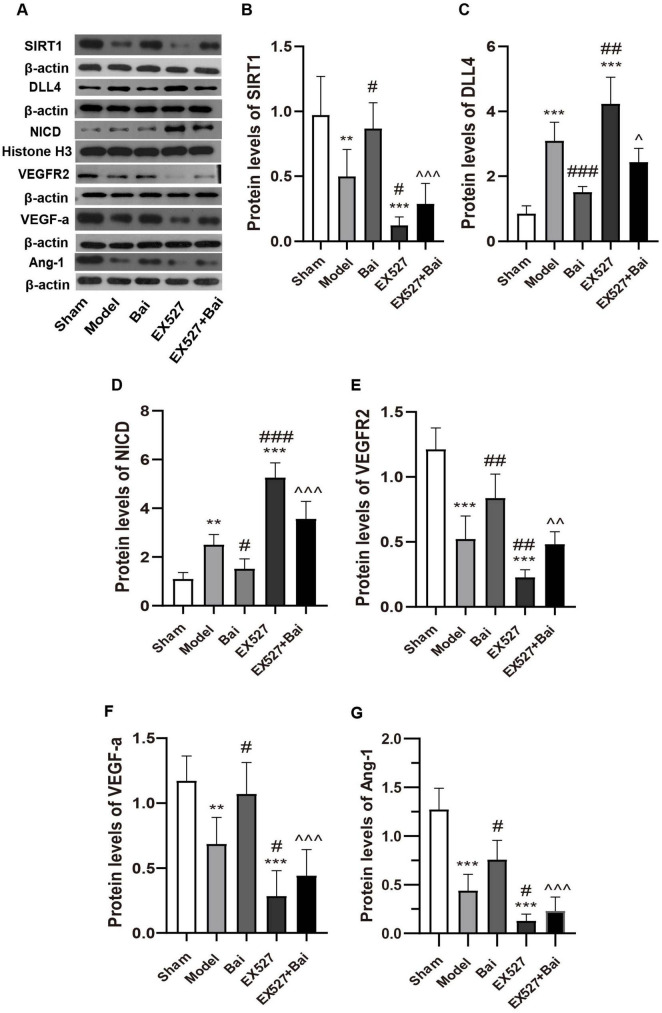
Baicalein regulates the SIRT1-mediated Notch1 signaling pathway and related angiogenic factors following CCH. **(A)** Western blot analysis of the expression of SIRT1, DLL4, NICD, VEGFR2, VEGF-a, and Ang-1 (*n* = 6 in each group). **(B)** Quantitative analysis of the protein levels of SIRT1. **(C)** Quantitative analysis of the protein levels of DLL4. **(D)** Quantitative analysis of the protein levels of NICD. **(E)** Quantitative analysis of the protein levels of VEGFR2. **(F)** Quantitative analysis of the protein levels of VEGF-a. **(G)** Quantitative analysis of the protein levels of Ang-1. β-actin was used as an internal control. **p < 0.01, and ***p < 0.001: vs. the sham group; ^#^*p* < 0.05, ^##^*p* < 0.01, and ^###^*p* < 0.001: vs. the model group; ^∧^*p* < 0.05, ^∧∧^*p* < 0.01, ^∧∧∧^*p* < 0.001: vs. the Bai group. The values are expressed as the mean ± SD. SIRT1, silent mating type information regulation 2 homolog 1. DLL4, Delta-like 4; NICD, intracellular domain of Notch; VEGFR2, vascular endothelial growth factor 2; VEGF-a, vascular endothelial growth factor a; Ang-1, angiopoietin-1.

To ascertain whether the SIRT1-mediated Notch1 pathway and its associated angiogenic factors are implicated in baicalein-regulated angiogenesis, we conducted western blotting to measure the protein levels of SIRT1, VEGF-a, DLL4, NICD, VEGFR2 and Ang-1 (Figures 6A-G). In accordance with previous research indicating that SIRT1 plays a role in angiogenesis by modulating Notch1 signaling (Zhu et al., 2021), the current findings demonstrated that SIRT1 expression was reduced while DLL4 and nuclear NICD expression was elevated in rats with CCH in comparison to the sham rats (SIRT1, *P* < 0.01; DLL4, *P* < 0.001; NICD, *P* < 0.01, model group vs. sham group) (Figures 6A-D). While the upregulation of the DLL4/NICD pathway was alleviated by baicalein treatment, which was accompanied by an increase in SIRT1 expression (SIRT1, *P* < 0.05; DLL4, *P* < 0.001; NICD, *P* < 0.05, model group vs. Bai group) (Figures 6A-D). Moreover, the inhibition of SIRT1 with EX527 resulted in a further upregulation of the DLL4/NICD pathway (DLL4, *P* < 0.01; NICD, *P* < 0.001, model group vs. EX527 group) (Figures 6A,C,D). Moreover, the suppressing effect of baicalein on the DLL4/NICD pathway was eliminated by EX527 (DLL4, *P* < 0.05; NICD, *P* < 0.001, Bai group vs. EX527+Bai group) (Figures 6A,C,D).

In accordance with the results of immunofluorescence staining, the downstream angiogenic factor of Notch1 (VEGFR2, VEGF-a and Ang-1) decreased in the model group (VEGF-a, *P* < 0.01; VEGFR2, *P* < 0.001; Ang-1, *P* < 0.001, model group vs. sham group) (Figures 6A,E-G) and in the EX527 group (all *P* < 0.001, EX527 group vs. sham group) (Figures 6A,E-G). The administration of baicalein was observed to mitigate the reduction in these angiogenic factors (VEGF-a, *P* < 0.05; VEGFR2, *P* < 0.01; Ang-1, *P* < 0.05, model group vs. Bai group) (Figures 6A,E-G), while inhibiting SIRT1 exacerbated the decline in these angiogenic factors (VEGF-a, *P* < 0.05; VEGFR2, *P* < 0.01; Ang-1, *P* < 0.05, model group vs. EX527 group) (Figures 6A,E-G) and partially eliminated the angiogenic effect of baicalein (VEGF-a, *P* < 0.001; VEGFR2, *P* < 0.01; Ang-1, *P* < 0.01, EX527+Bai group vs. Bai group) (Figures 6A,E-G). These results substantiate the hypothesis that baicalein promotes angiogenesis by regulating the SIRT1-mediated Notch1 Signaling.

## 4 Discussion

Recent investigations have revealed that therapeutic strategies that stimulate angiogenesis can ameliorate vascular cognitive impairment (Ren et al., 2018; Wang and Hu, 2018). The objective of the present study was to evaluate the effect of baicalein on angiogenesis in a rat model of VD and to identify its potential therapeutic targets. The findings indicated that baicalein mitigated cognitive impairment, promoted angiogenesis and suppressed neuroinflammation and apoptosis by regulating the SIRT1-mediated Notch1 pathway. Moreover, to the best of our knowledge, this is the inaugural investigation to implicate the SIRT1-mediated Notch1 pathway in angiogenesis in the context of VD.

It is well established that the brain requires a continuous and well-regulated supply of blood due to its high energy demands and limited fuel reserves. Chronic hypoperfusion resulting from high-grade stenosis or occlusion of the carotid arteries has been demonstrated to cause cognitive impairment in both clinical and animal experiments (Bhandari et al., 2024; Niu et al., 2019). Angiogenesis plays a pivotal role in restoring cerebral blood flow (CBF) in vascular disease and has been identified as a key target for therapeutic intervention (Ren et al., 2018). Indeed, therapeutic approaches that stimulate angiogenesis have been demonstrated to enhance cognitive restoration, which was compromised when angiogenesis was obstructed in VD models (Liu et al., 2017; Ren et al., 2018; Sathyamoorthy et al., 2019). The processes of angiogenesis and neural activity are closely interrelated. Following cerebral ischemia-induced injury, neuroblasts consistently congregate in proximity to blood vessels (Yamashita et al., 2006). Angiogenic factors such as Ang-1 and VEGF are partly derived from neurogenic secretion (Chen et al., 2009; Li et al., 2013). In the present study, we employed immunofluorescence staining of CD31 to assess MVD, which is regarded as a practical parameter for the quantitative assessment of angiogenesis (Zhang et al., 2018), and double immunofluorescence labeling of CD31 and BrdU to evaluate endothelial cell proliferation. The results showed that the MVD decreased in rats with CCH, which is consistent with the results of previous studies (Niu et al., 2021; Xiong et al., 2017). Angiogenesis serves as a compensatory response to hemodynamic changes. Hypoxia-inducible factor 1 (HIF-1), a pivotal transcription factor in the endogenous adaptive response, is induced by conditions of hypoxia or ischemia in stroke and CCH (Wang et al., 2019; Yang et al., 2017) and it has been shown to promote the expression of genes associated with angiogenesis (Correia and Moreira, 2010). This may provide a molecular basis for the increased endothelial cell proliferation observed in rats with CCH. The results also demonstrated that rats treated with baicalein exhibited enhanced angiogenesis and superior cognitive function compared to untreated rats with CCH. It can be postulated that baicalein improves cognitive function following CCH, at least in part, by promoting angiogenesis.

The connection between angiogenesis and neuroinflammation and subsequent apoptosis is of significant importance in determining the fate of cells. For example, HIF-1 and its downstream factor VEGF facilitate angiogenesis along with neovascularization in ischemia-induced VECs injury at the later phase of acute ischemia (Liu et al., 2018), promote glucose inflow into astrocyte to maintain ATP levels and cell survival (Jimenez-Blasco et al., 2020), increase capillary permeability to go through the endothelium-mesenchymal transformation, which causes BBB disruption and subsequent neuroinflammation and apoptosis (Chen et al., 2019; Zhang et al., 2016), mediate microglia polarization and the expression of microglia-associated pro-inflammatory factors (Xu et al., 2020), exacerbate neuronal damage by upregulating the level of TLR4 (Yao et al., 2013b), and activate NLRP3 in microglia (Jiang et al., 2020) at the acute phase of ischemia/reperfusion. In order to elucidate the relationship between angiogenesis and neuroinflammation and subsequent apoptosis in CCH, the present study employed immunofluorescence staining and ELISA to detect the changes of MVD, astrocytes, microglia and pro-inflammatory factors. The results demonstrated a reduction in MVD in rats with CCH, accompanied by astrocyte and microglia proliferation and elevated levels of TNF-α and IL-1β. However, treatment with baicalein resulted in an increase in MVD and CD31+/BrdU+ cells, accompanied by a reduction in neuroinflammation and apoptosis. These all contributed to cognitive function recovery, and SIRT1 was identified as a key factor in this process.

To date, the molecular basis underlying angiogenesis and neuroinflammation in CCH remains incompletely elucidated. The involvement of SIRT1/VEGF signaling in the regulation of angiogenesis and neuroinflammation is well established (Li et al., 2020; Lin et al., 2018; Yang et al., 2022; Zheng et al., 2018). SIRT1, an NAD (+)-dependent deacetylase, is highly expressed in the vasculature during the process of angiogenesis and plays a role in controlling the sprouting and branching activity of endothelial cells (Zhang et al., 2020). Silencing SIRT1 abolishes endothelial sprout formation and results in defective vascular growth and patterning (Wang et al., 2014). Mice with SIRT1 gene knockout are more vulnerable to ischemic stress and exhibit larger infarct volumes (Hernández-Jiménez et al., 2013). Conversely, strategies that promote SIRT1 expression have been demonstrated to relieve hippocampal neuron damage and cognitive impairment following CCH (Li et al., 2020). VEGF is a critical regulator of angiogenesis under both physiological and pathological conditions and is also recognized as a confirmed downstream target of SIRT1 (Kudo et al., 2018; Zheng et al., 2018). VEGF is a key initiator of sprouting during angiogenesis, while Ang-1 contributes to the maturation and remodeling of the immature vasculature (Fagiani and Christofori, 2013). Previous studies have demonstrated that pharmacological promotion of SIRT1/VEGF signaling contributes to angiogenesis following ischemic brain injury (Mi et al., 2019; Zheng et al., 2018). Moreover, SIRT1/VEGF has been shown to inhibit the proliferation of glial cells and the expression of pro-inflammatory factors (Boisserand et al., 2024; He et al., 2021; Li et al., 2020). Additionally, SIRT1/VEGF is also critical to brain cognition, with evidence suggesting that inhibiting VEGF can lead to cognitive impairment (Koester-Hegmann et al., 2018; Li et al., 2020; Ng et al., 2014). Consistent with these findings, the present study demonstrated that in the early stage of CCH, the expression of SIRT1, Ang-1 and VEGF was down-regulated in rats with CCH, indicating a pathological response. This change was accompanied by reduced MVD and activated neuroinflammation and apoptosis. Conversely, rats treated with baicalein exhibited higher levels of SIRT1, VEGF-a and Ang-1, as well as enhanced angiogenesis and decreased neuroinflammation and apoptosis. Moreover, the enhancement of angiogenesis and the suppression of neuroinflammation and apoptosis can be negated by the inhibition of SIRT1, indicating that the neuroprotective effect of baicalein is achieved by the modulation of the SIRT1 pathway.

Notch signaling is of critical importance in the determination of cell fate in a multitude of cell types, both during the developmental period and in postnatal life. In growing blood vessels, the endothelial sprouting is inhibited by Notch signaling. This inhibition is mediated by the binding of the Notch receptor to its ligand DLL4. Once bound, DLL4 triggers the proteolytic release of NICD, which translocates into the nucleus to activate the transcription of downstream target genes, resulting in a reduction of VEGFR2 expression and low endothelial cell sensitivity to VEGF (Wang et al., 2014). Moreover, previous studies have demonstrated that Notch1 enhances IL-1β expression and elevates endothelial permeability, thereby precipitating endothelial dysfunction in human brain microvascular endothelial cells (Park et al., 2021). Genetic knockout of Notch1 has been demonstrated to result in a reduction in macrophage infiltration, proinflammatory mediators and apoptosis (Yang et al., 2023). It has been demonstrated that inhibiting the Notch signaling results in a reduction of NF-kB/p65 translocation, which occurs through the suppression of the TLR4/ MyD88/TRAF6 pathway (Yao et al., 2013a). The present study demonstrated that rats with CCH exhibited elevated levels of Notch1 pathway-related molecules, accompanied by reduced MVD and heightened neuroinflammation and apoptosis compared to the sham rats. The baicalein-treated rats showed a reduced level of Notch1 pathway-related molecules, enhanced angiogenesis, and a reduction in neuroinflammation and apoptosis. These opposing changes have implicated for the first time the role of Notch1 in regulating angiogenesis and neuroinflammation in VD.

The relationship between SIRT1 and the Notch1 pathway is characterized by a high degree of complexity at the regulatory level. SIRT1 deacetylates NICD, which results in the inhibition of NICD translocation into the nucleus and subsequent inhibition of downstream factors, including Hes1 and Hey1. This leads to the upregulation of VEGFR2 and VEGFR3 in endothelial progenitor cells (Wang et al., 2014). Another study demonstrated that the expression of DLL4, Hey1, Hes1 and NICD was elevated in microvascular endothelial cells in SIRT1 mutant mice (Kida et al., 2016). In consistent with the aforementioned findings, the present study demonstrated that the inhibition of SIRT1 resulted in the overactivation of the Notch1 pathway, accompanied by a heightened degree of neuroinflammation and apoptosis, and a reduction in angiogenesis. Treatment with baicalein up-regulates the expression of SIRT1 and down-regulates the Notch1 pathway, thereby promoting angiogenesis and suppressing neuroinflammation and apoptosis. This study presents the inaugural evidence that SIRT1 functions as a mediator of Notch1 signaling, thereby promoting angiogenesis and inhibiting neuroinflammation and apoptosis in the context of VD. Baicalein exerts a cognitive protective effect by regulating the SIRT1-mediated Notch1 pathway.

Previous studies have yielded controversial conclusions regarding the relationship between baicalein and angiogenesis. Baicalein strengthens angiogenesis and increases blood flow to ischemic limbs in diabetic mice (Liu et al., 2023), but inhibits angiogenesis and synovial proliferation to alleviate osteoarthritis (Li et al., 2021). The present study offers novel evidence that baicalein exerts the capacity to both promote angiogenesis and attenuate neuroinflammation and apoptosis in rats following CCH. In previous studies by our team, baicalein promoted remyelination (Xiao et al., 2023), suppressed neuroinflammation and regulated the composition of the intestinal microbiota (Song et al., 2024). These effects collectively contribute to a cognitive protective effect. The present findings further substantiate this effect by indicating angiogenesis. Consequently, the therapeutic mechanism of baicalein in VD is rendered more plausible, thereby expanding the potential applications of baicalein in VD.

It should be noted that this study is subject to a number of limitations. Firstly, the sample sizes were relatively small. Secondly, the assessment of outcomes was conducted at a single time point post-surgery (4 weeks post-surgery) due to the observation of severe pathological changes in angiogenic factors at this time point, as evidenced by previous studies and preliminary experiments (Li et al., 2020; Xiong et al., 2017).

## 5 Conclusion

**FIGURE 7:**
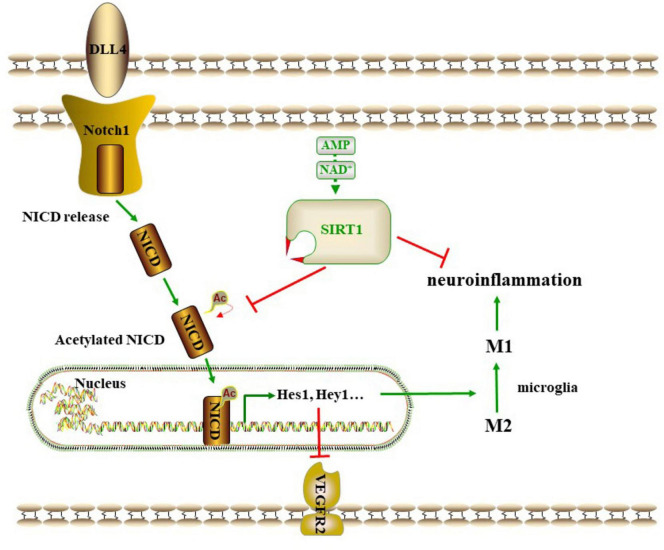
A proposed scheme on the SIRT1 -mediated Notch1 pathway.

In conclusion, the present findings demonstrated that baicalein is able to alleviate cognitive deficiency in rats with CCH by promoting angiogenesis and suppressing neuroinflammation and apoptosis through a mechanism involving the regulation of the SIRT1-mediated Notch1 pathway (Figure 7). Baicalein may serve as a promising agent for the promotion of angiogenesis, the suppression of neuroinflammation and the protection of cognitive function in VD. The SIRT1-mediated Notch1 pathway may represent a potential target for future therapies. Further studies are needed to explore the deeper mechanisms underlying the protective effect of baicalein in the context of VD.

## Data Availability

The raw data supporting the conclusions of this article will be made available by the authors upon request.
