# Supplementary figures and images for: Baicalein ameliorates cognitive decline induced by chronic cerebral hypoperfusion through the SIRT1-mediated Notch1 pathway to improve angiogenesis and suppress neuroinflammation
Source: Front Aging Neurosci. 2025 Apr 11;17:1521353. doi: 10.3389/fnagi.2025.1521353 (PMC12021866; doi:10.3389/fnagi.2025.1521353)

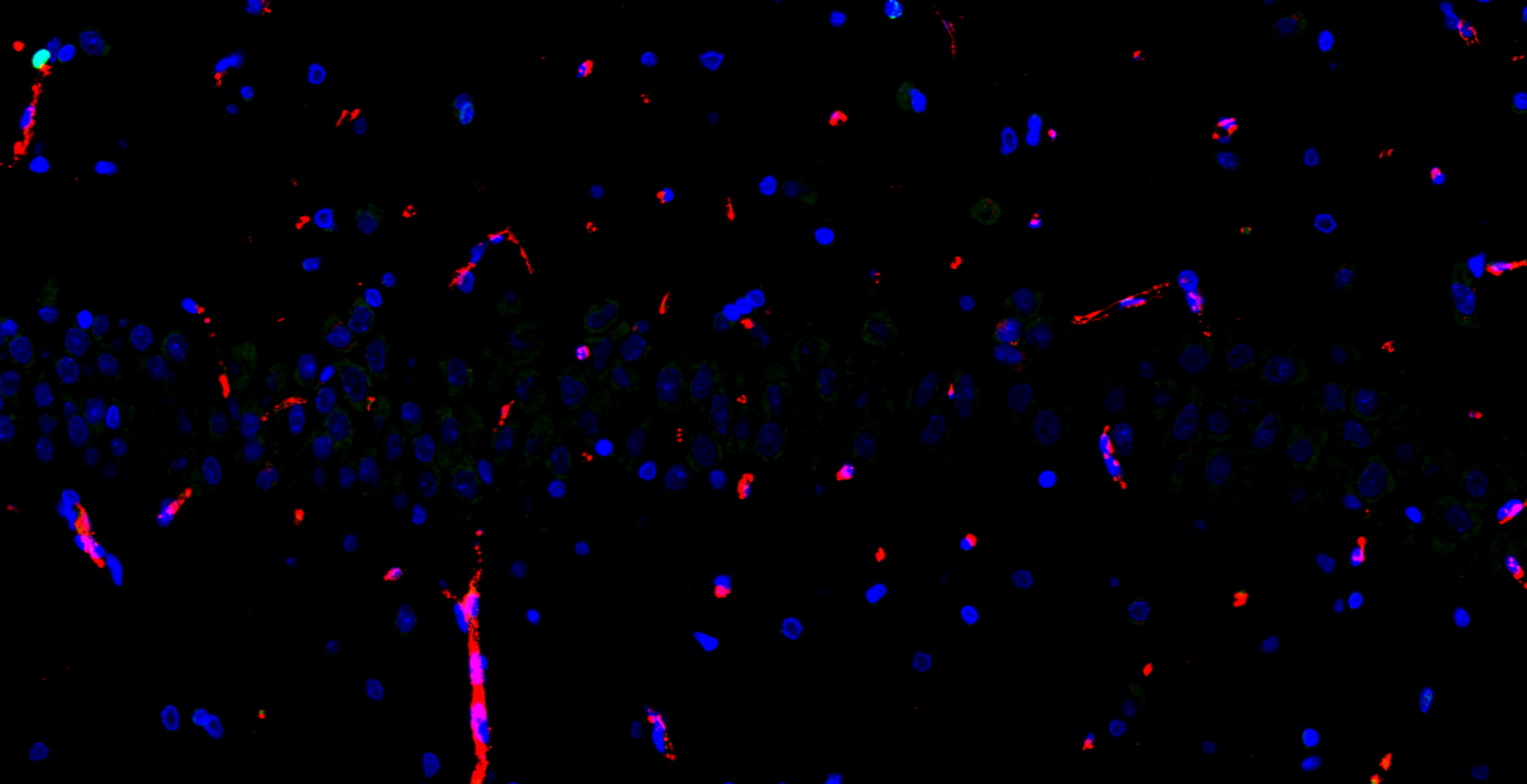















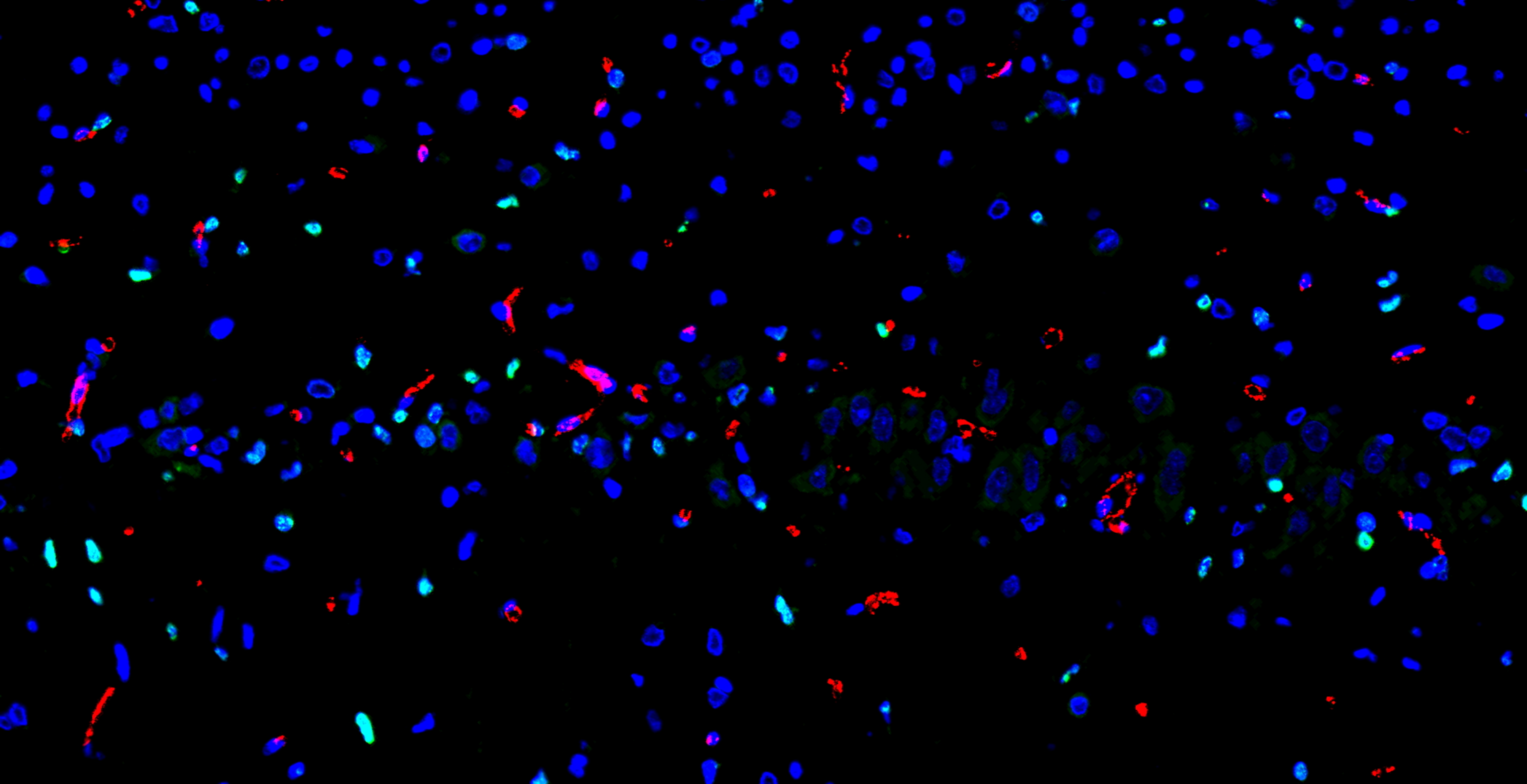





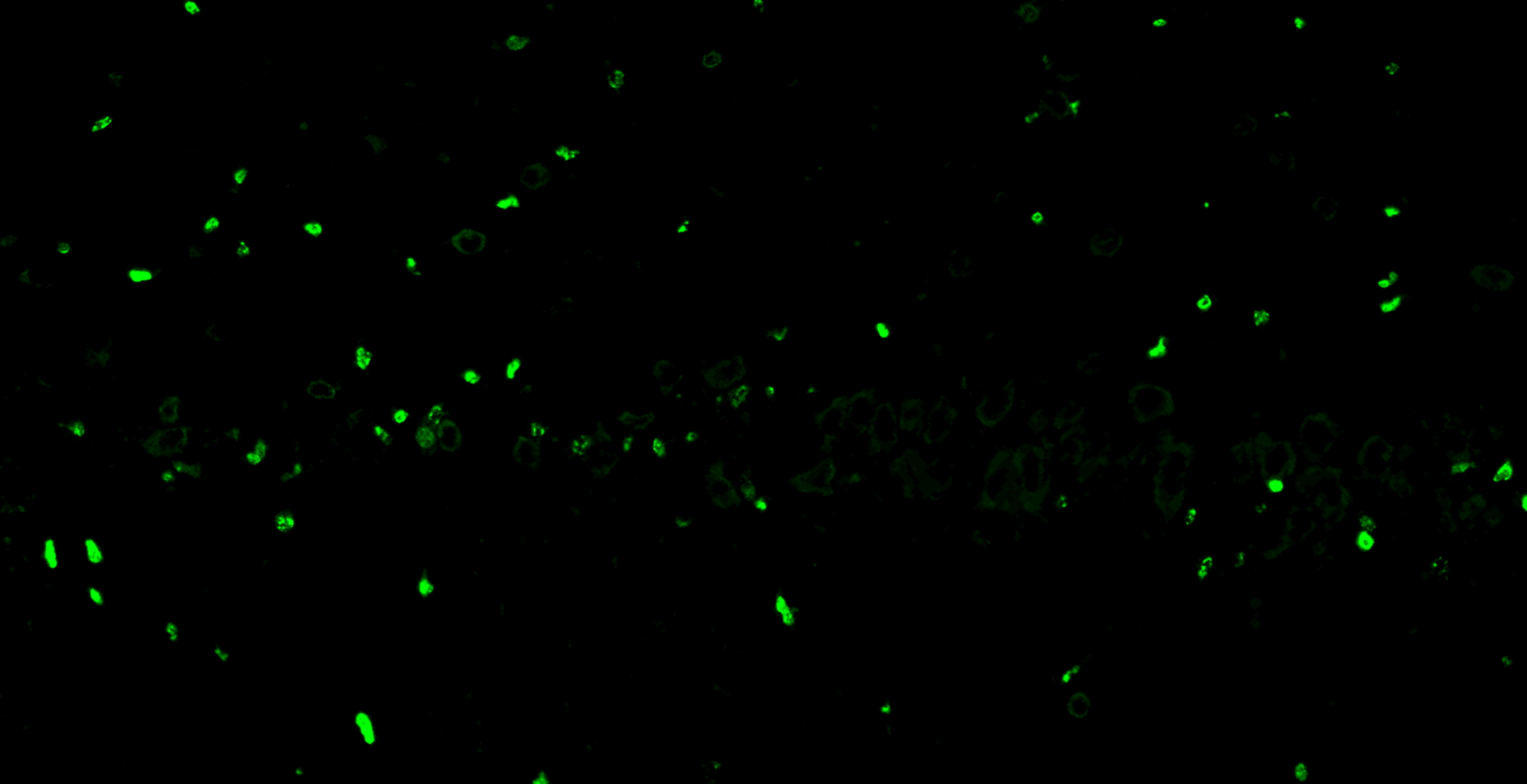

















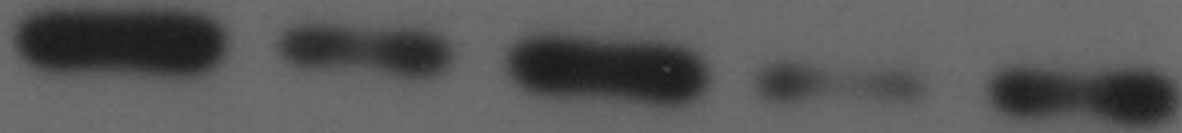

CD31

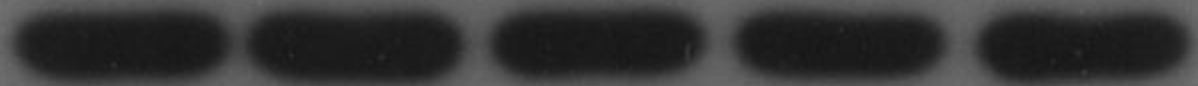

$\beta$ -actin

Supplement: Supplementary file 1 [file Data_Sheet_1.pdf]

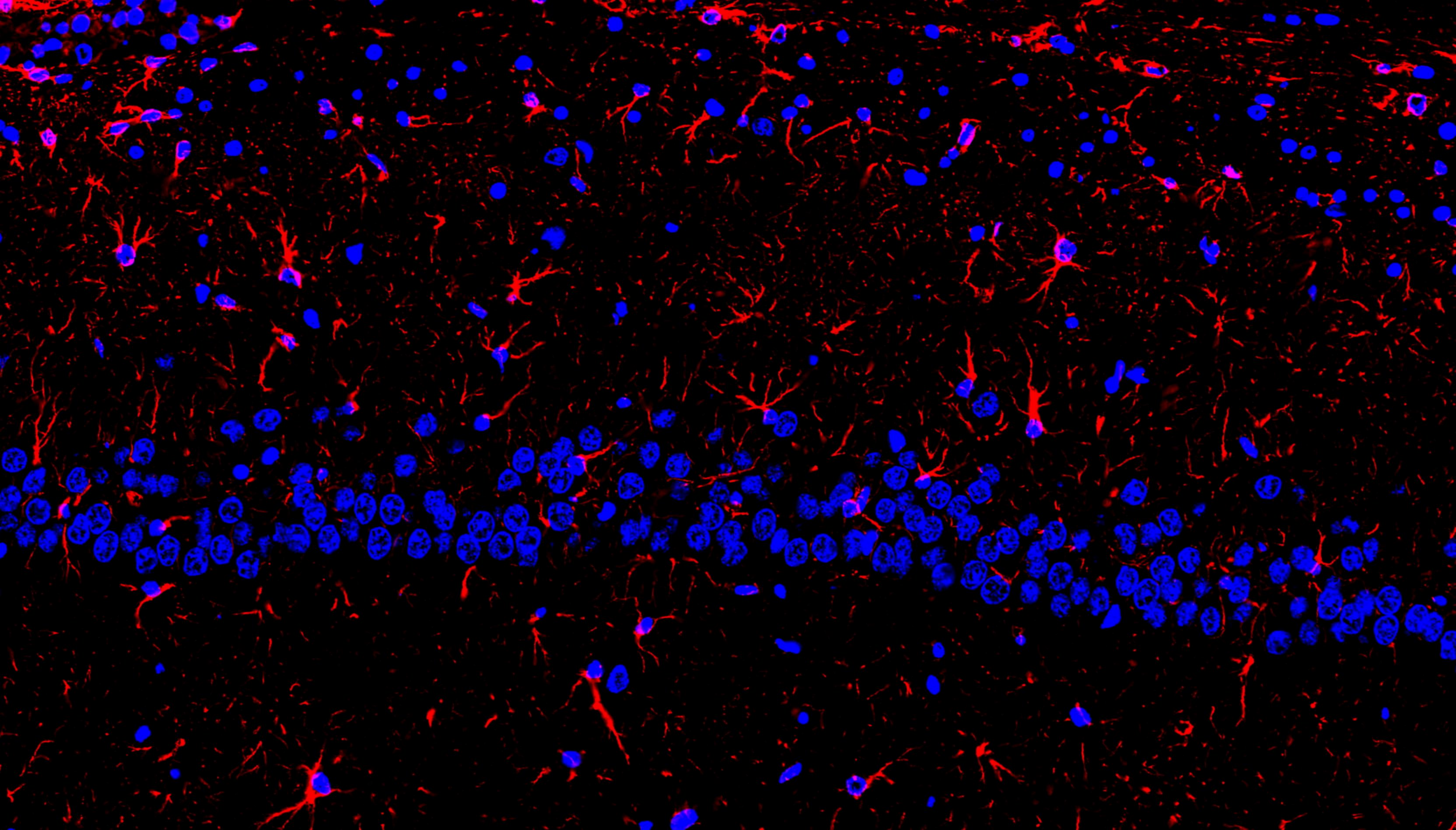

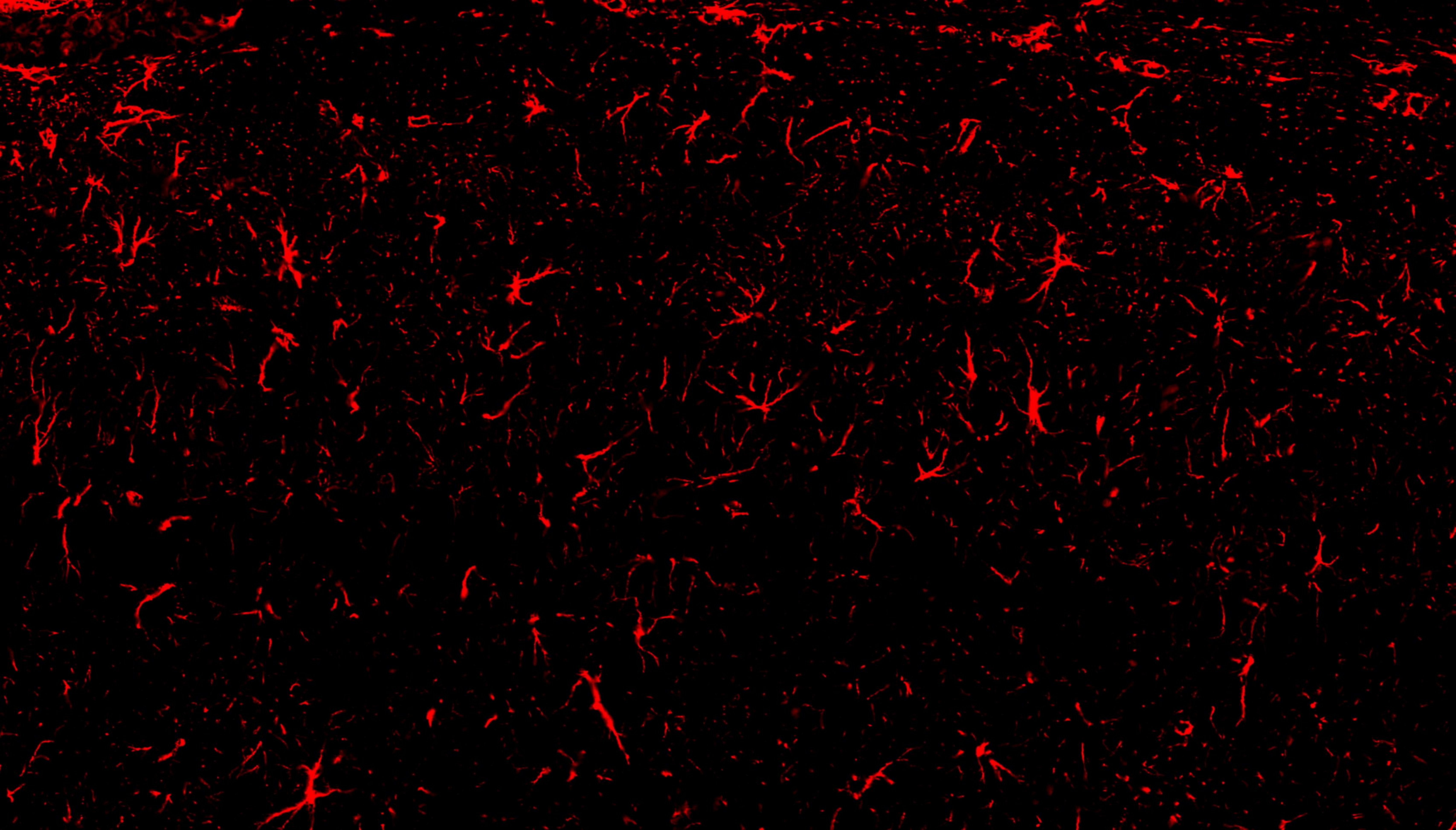





















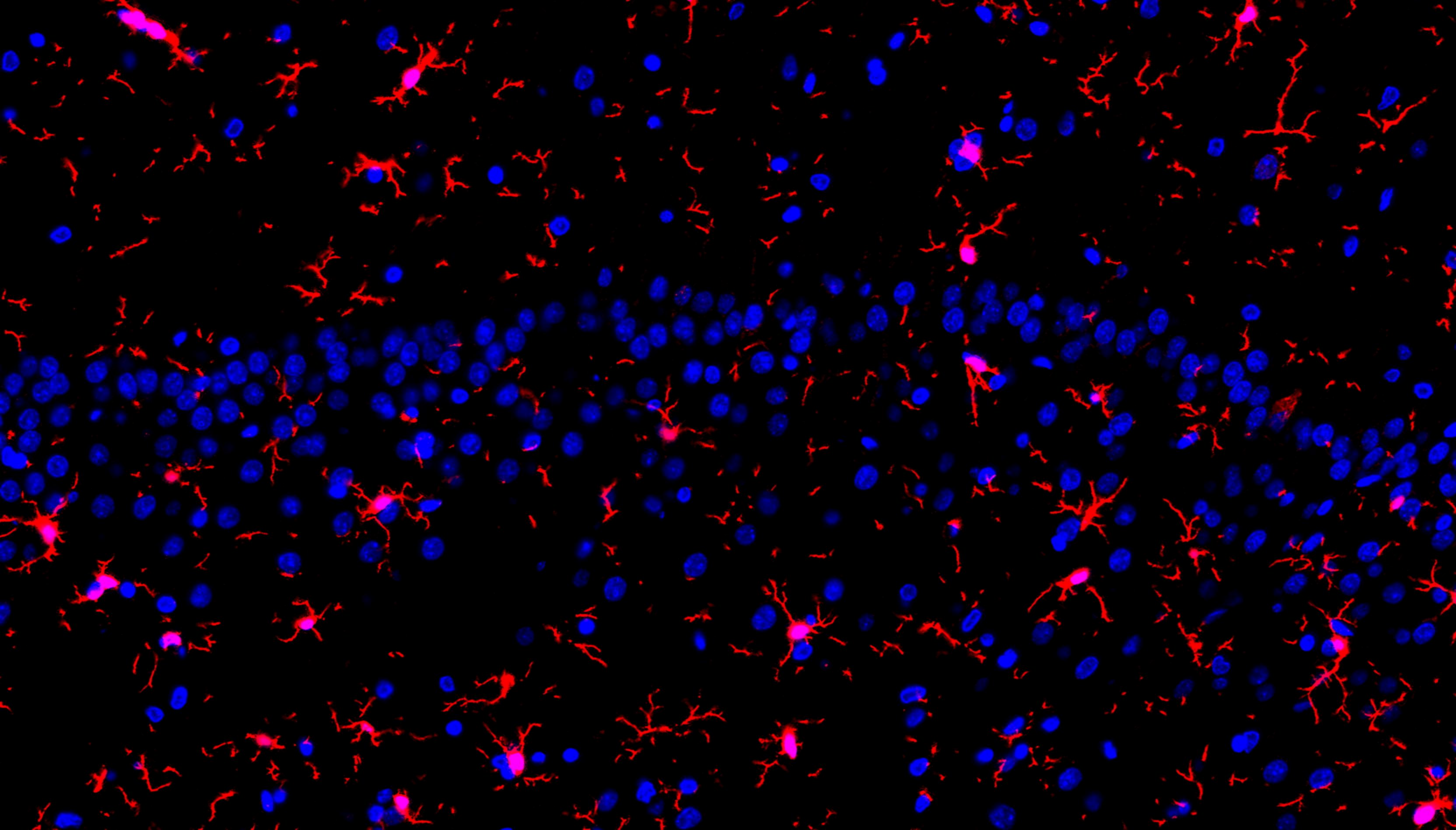

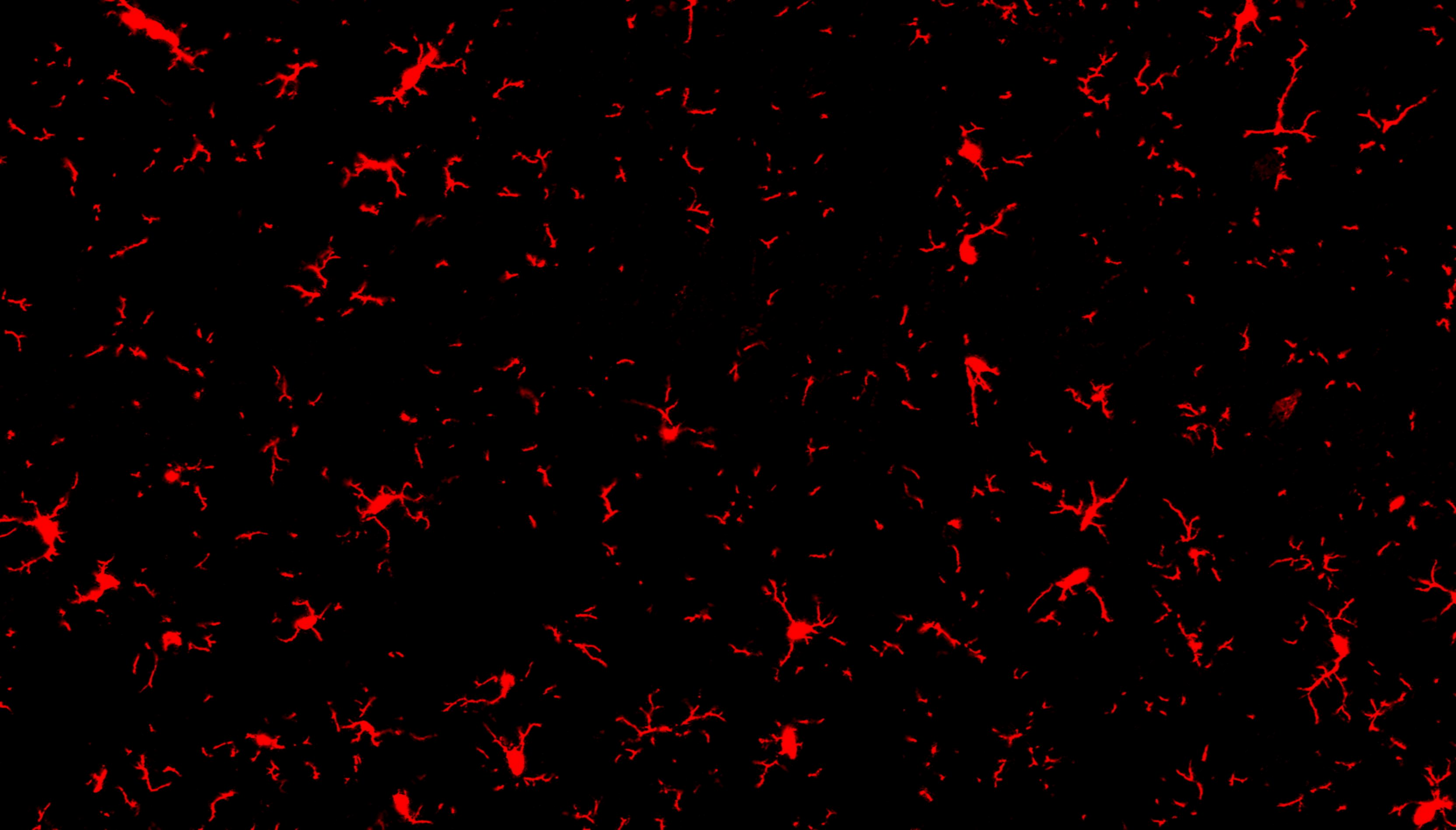

Supplement: Supplementary file 2 [file Data_Sheet_2.pdf]

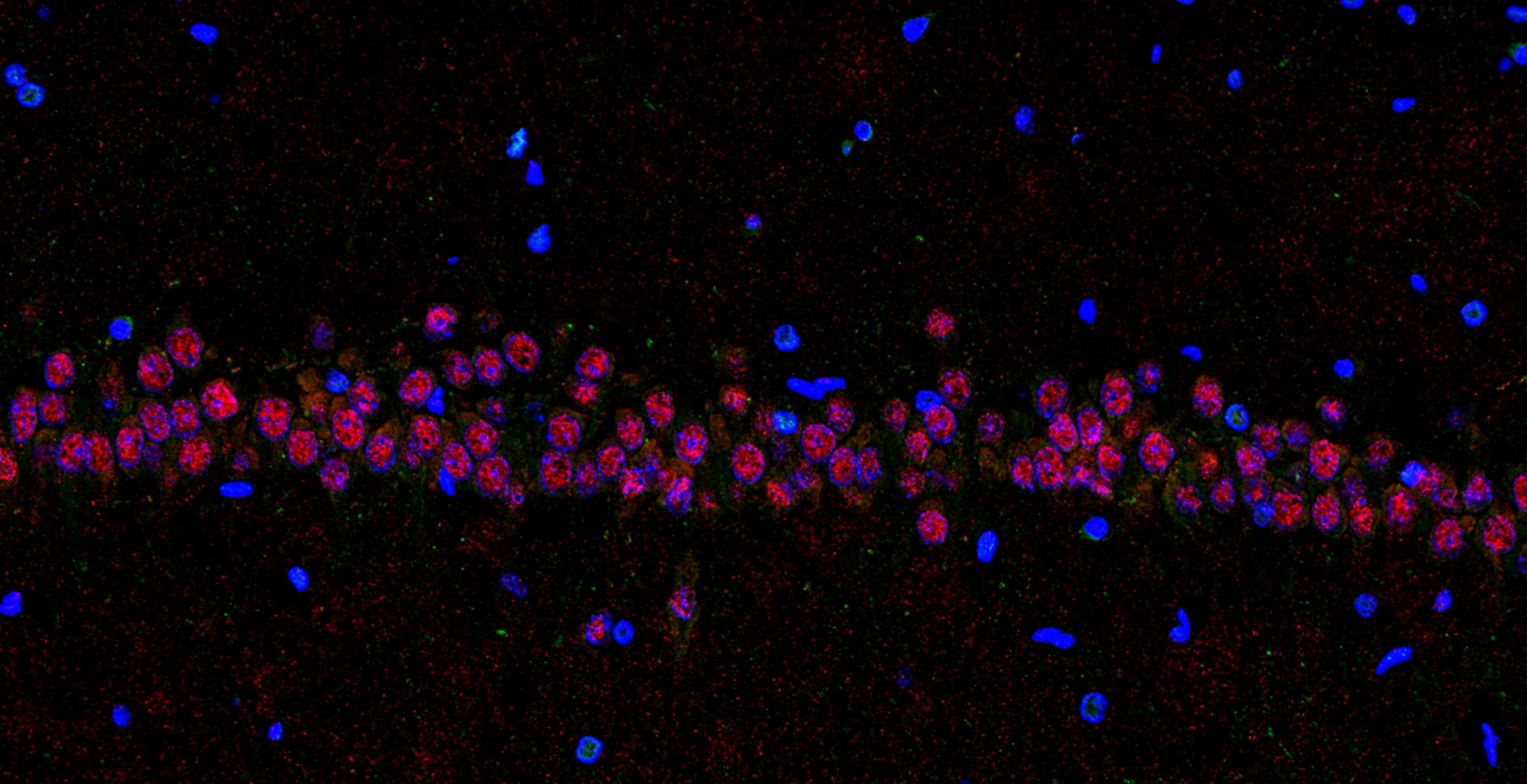







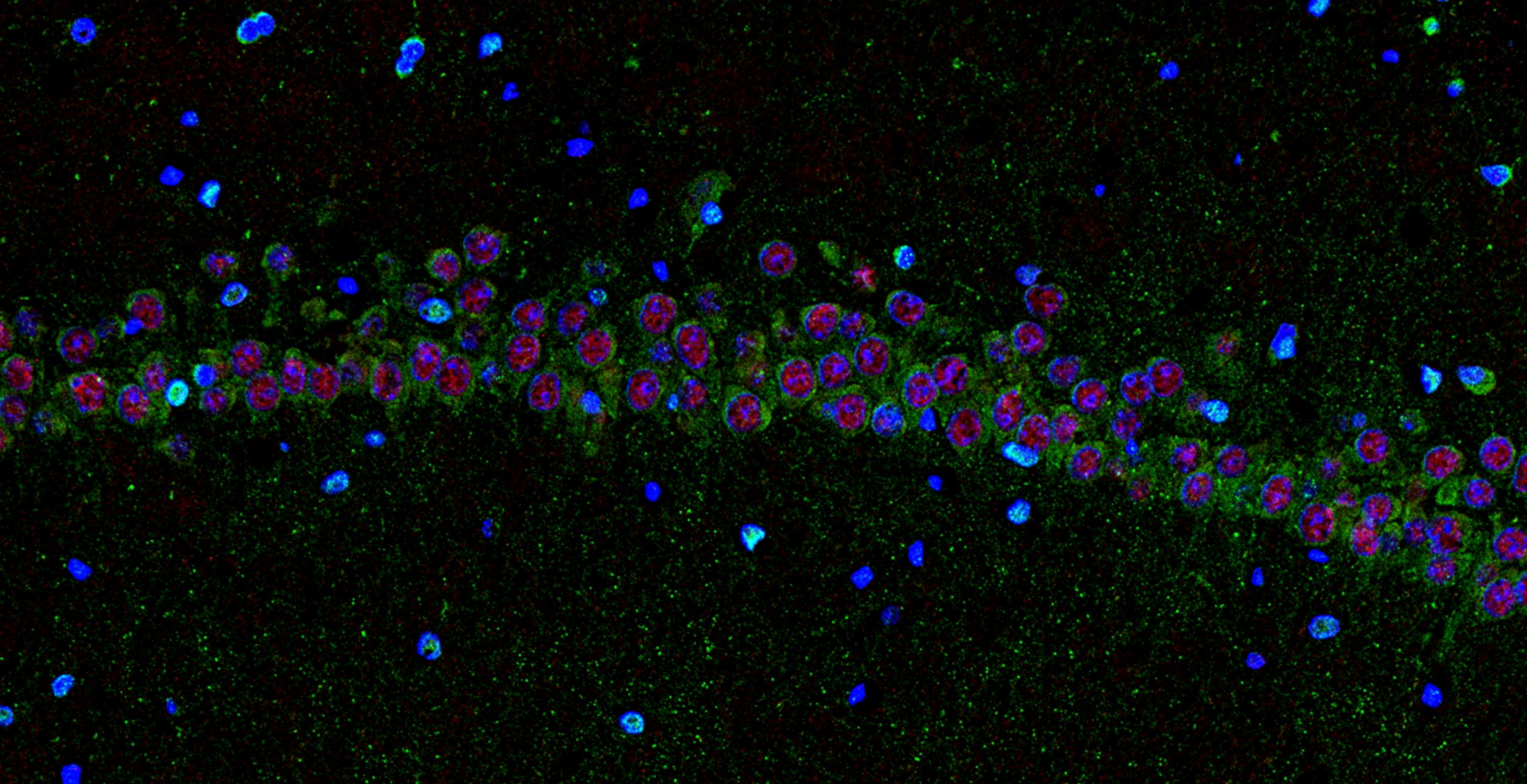





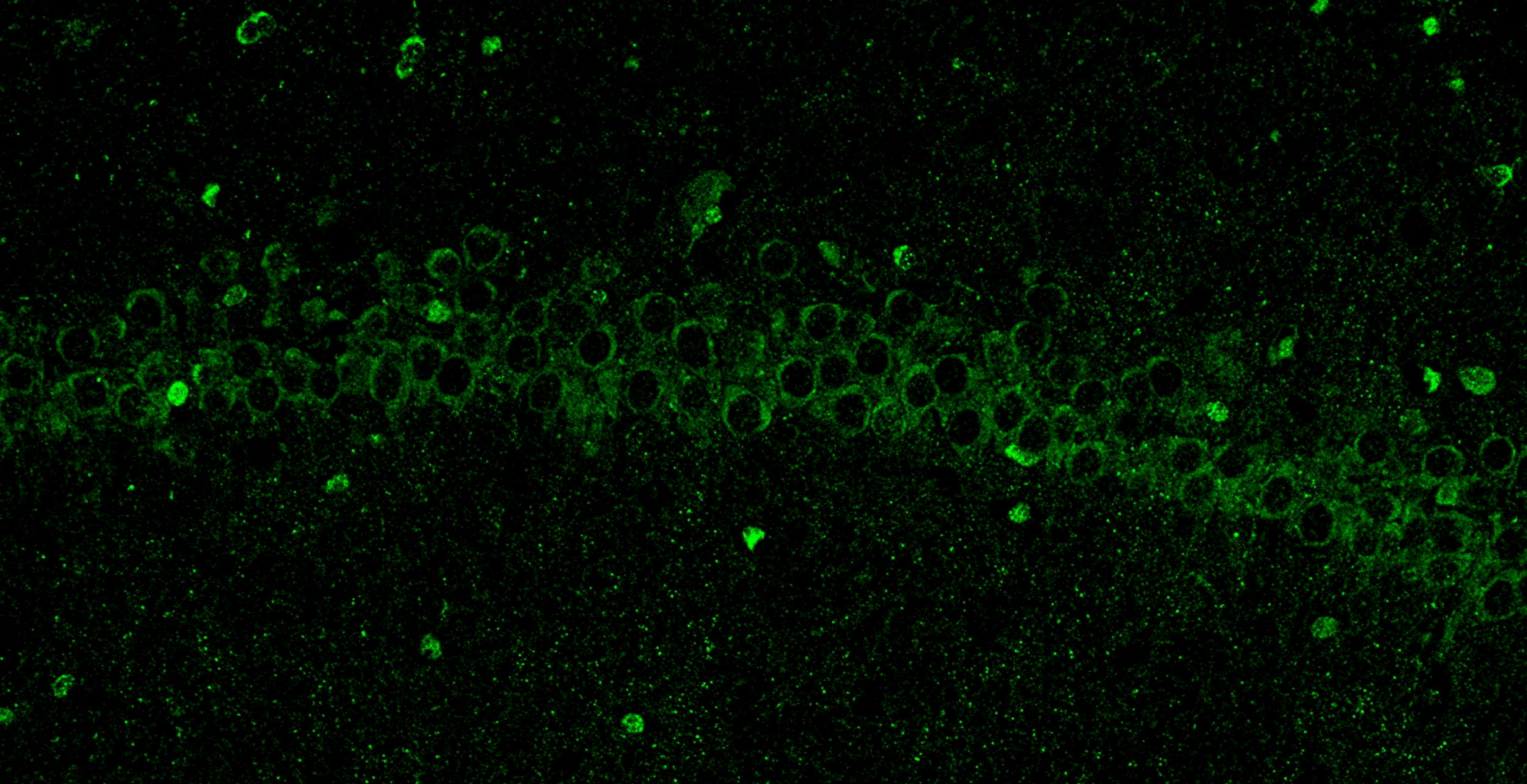









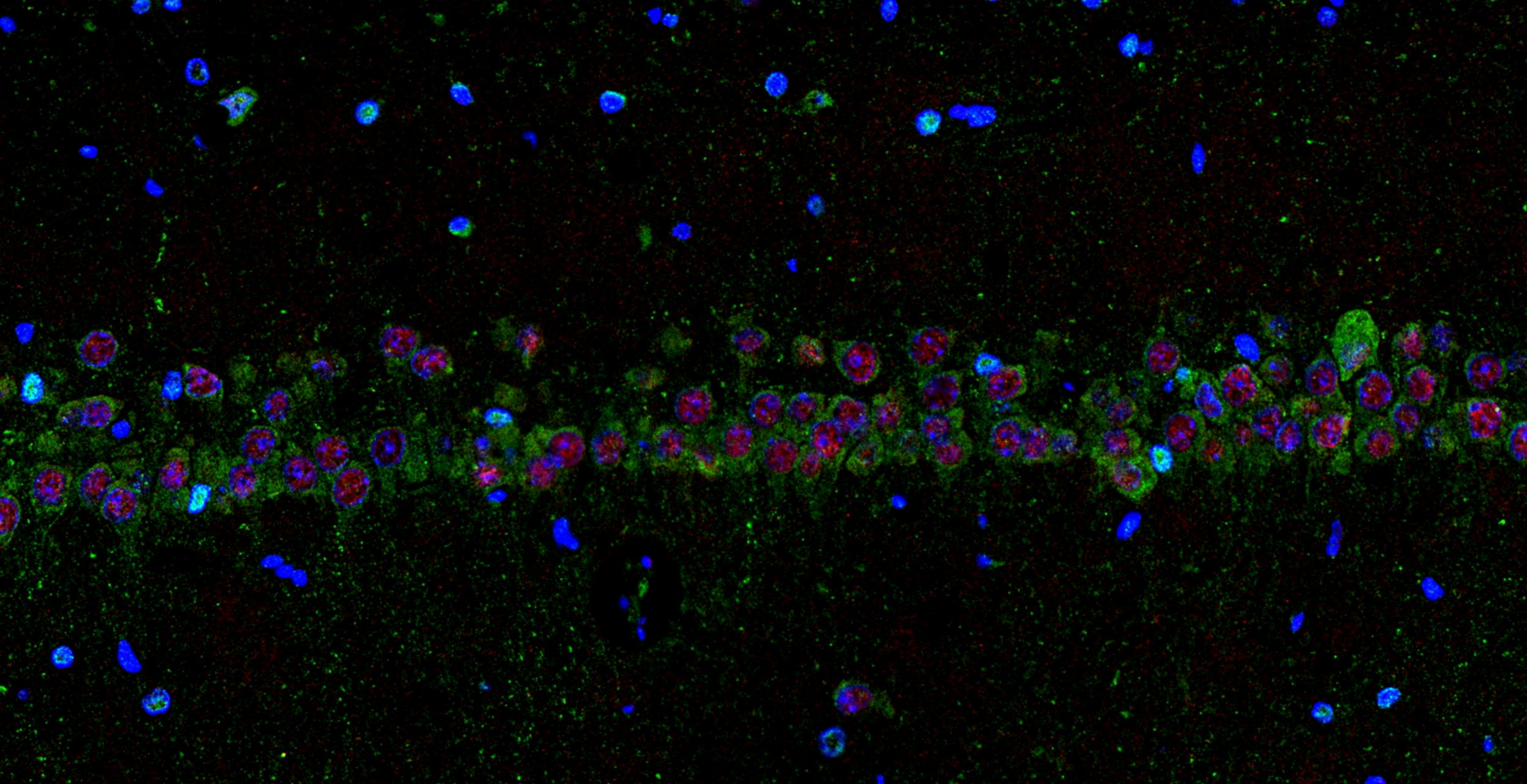





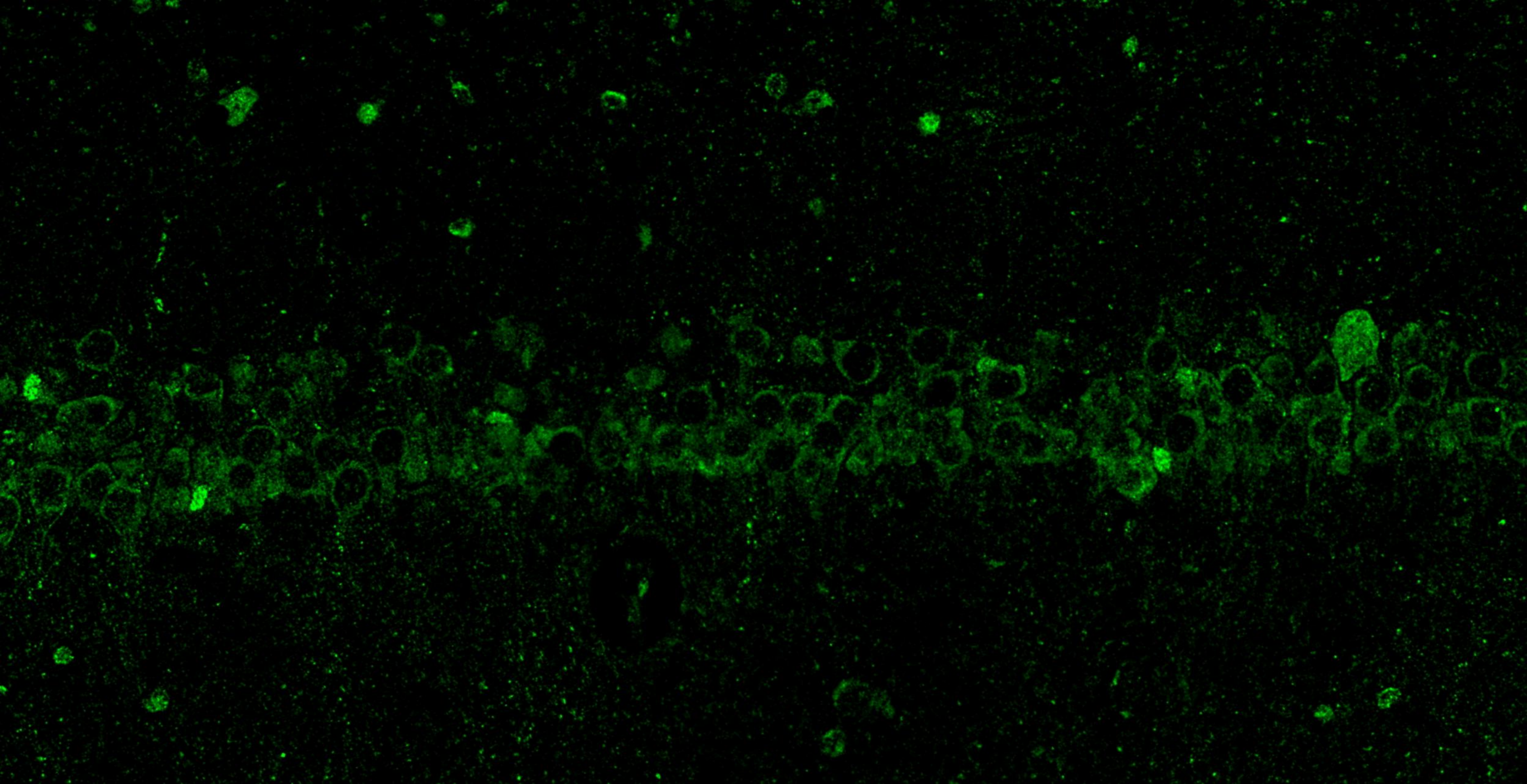

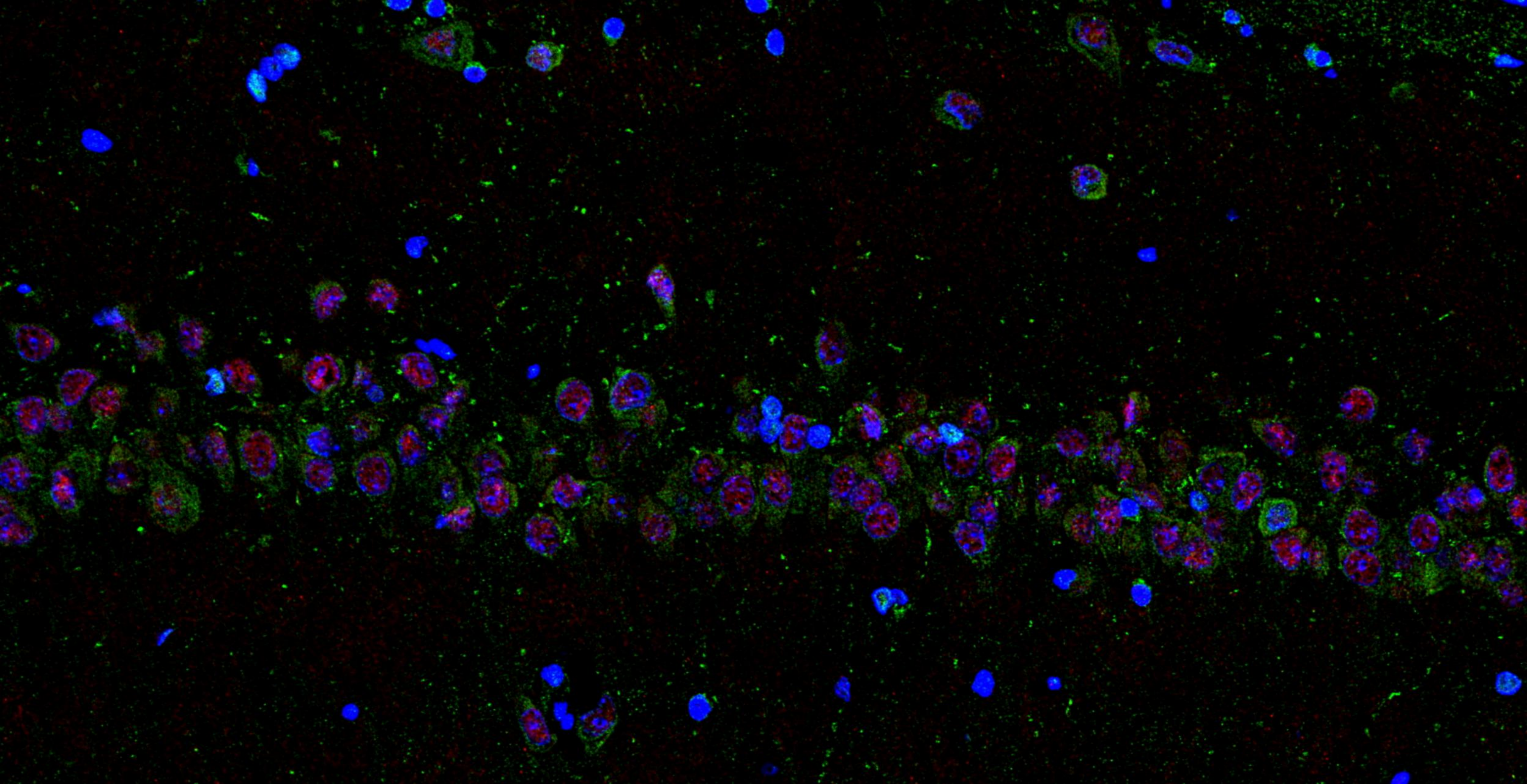







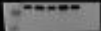

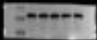

Supplement: Supplementary file 3 [file Data_Sheet_3.pdf]

1st

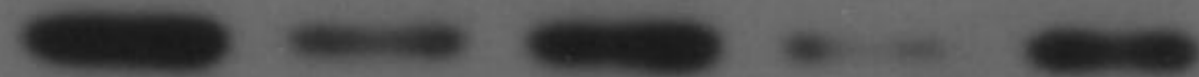

AKC12

55-

40-

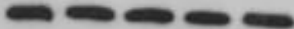

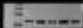

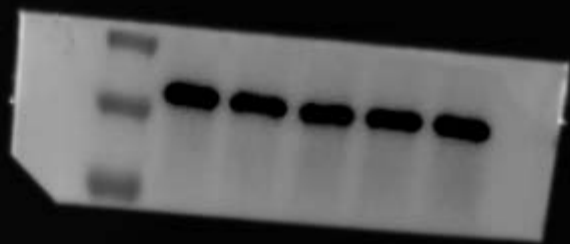

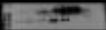

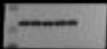

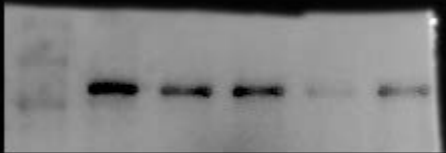

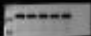

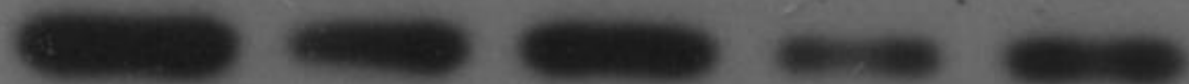

VEGF

Actin 3

52 -

40 -

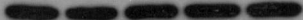

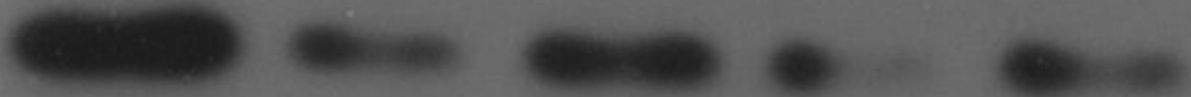

ang!

Actin

55

40

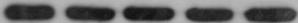

Supplement: Supplementary file 4 [file Data_Sheet_4.pdf]
